# Differential Morphophysiological and Biochemical Responses of Cotton Genotypes Under Various Salinity Stress Levels During Early Growth Stage

**DOI:** 10.3389/fpls.2021.622309

**Published:** 2021-03-11

**Authors:** Wajeeha Munawar, Amjad Hameed, Muhammad Khashif Riaz Khan

**Affiliations:** Nuclear Institute for Agriculture and Biology College, Pakistan Institute of Engineering and Applied Sciences, Faisalabad, Pakistan

**Keywords:** salt stress, antioxidants, salinity, SOD, CAT, POD, cotton seedlings

## Abstract

Cotton is a primary agriculture product important for fiber use in textiles and the second major oil seed crop. Cotton is considered as moderately tolerant to salt stress with salinity threshold of 7.7 dS/m at seedling stage. Salinity causes reduction in the growth of seedlings and cotton production that limits fiber quality and cotton yield. In this study, initially, 22 cotton genotypes were screened for relative salt tolerance using germination test in Petri plates (growth chamber). Selected 11 genotypes were further tested in pot experiment (sand) with 0, 15, and 20 dS/m NaCl treatments under glass house conditions. At four-leaves stage, different morphological and physiological traits were measured for all genotypes while biochemical analysis was performed on selected seven highly tolerant and sensitive genotypes. NaCl treatment significantly reduced plant biomass in two genotypes IR-NIBGE-13 and BS-2018, while NIAB-135, NIAB-512, and GH-HADI had least difference in fresh weight between the control and NaCl-treated plants. Photosynthetic rate was maintained in all the genotypes with the exception of SITARA-16. In two sensitive genotypes (IR-NIBGE-13 and 6071/16), Na^+^ ion accumulated more in leaves as compared to K^+^ ion under stress conditions, and an increase in Na^+^/K^+^ ratio was also observed. The lesser accumulation of malondialdehyde (MDA) content and higher activity of enzymatic antioxidants such as superoxide dismutase (SOD), peroxidase (POD), and ascorbate peroxidase (APX) in stressed plants of NIAB-135, NIAB-512, and FH-152 indicated that these genotypes had adaption capacity for salinity stress in comparison with sensitive genotypes, i.e., IR-NIBGE-13 and 6071/16. The observed salt tolerance was corelated with plant biomass maintenance (morphological), photosynthetic rate, and ionic homeostasis (K^+^/Na^+^ ratio, physiological) and biochemical stress marker regulations. After a series of experiments, it was concluded that NIAB-135, NIAB-512, and FH-152 could be utilized in breeding programs aimed at improving salinity tolerance in cotton and can expand cotton cultivation in saline area.

## Introduction

Soil salinization is rapidly increasing day by day and became a global environmental problem (Ahammed et al., 2018). It decreases average yields of most major crops like wheat, rice, and cotton over and above 50% on a global scale (Bartels and Sunkar, 2005). The excessive concentration of salt (above from threshold, i.e., 3–4 dS/m) in the soil, water, and plant is called salinity (Hussain et al., 2019). Exposure to salt stress triggers many adverse physiological and biochemical changes in plants leading to yield reduction. Currently, out of 230 m ha of irrigated land, 45 m-ha area is under the influence of salt (Athar and Ashraf, 2009). Cotton is a primary agriculture crop that covered the biggest textile manufacturing industries devising a strong yearly influence on country economic value of $600 billion all over the world (Jabran et al., 2019). The cotton fiber consumption is increasing as human population grows. *Gossypium hirsutum* (90%) (high-yielding characteristics and early cultivation system, Campbell et al., 2010) and Gossypium *barbadense* (8%) (extralong staple fiber source, Wang et al., 2015) play a very important role in cotton production due to their high yield potential and competitive benefit to cotton textiles producers (Hu et al., 2019). According to the 2019 report survey, India, China, United States, Pakistan, Brazil, Australia, Uzbekistan, and Turkey are included in the list of top cotton-producing countries (Ali et al., 2019). Although Pakistan is placed among the globally top 5 cotton-producing countries, on the other hand, its yield lags behind other top most countries due to low yield per unit area and increasing cotton import. However, cotton is proposed as a medium salt-tolerant crop with salinity threshold of 7.7 dS/m (Maas and Hoffman, 1977). However, low yield, poor plant growth, and germination are the main constraints that are affected by salinity and alkalinity of soil that limit cotton growth at the early stages of development (Ashraf and Ahmad, 2000; Bolek, 2010).

Cotton seed germination is an important phase, but unfortunately, it is also a very sensitive stage for harsh climate conditions. Salinity can hinder seed germination by reducing plant water uptake ability, impose drought to the plant, and deploy it from nutrients by disturbing the ions uptake mechanism (Wang et al., 2011). In salt stress condition, the plant undergoes cellular injury that occurs in transpiring leaves because of large amount of salt accumulation (Khan et al., 1995). It is also considered that the growth rate is directly related with stomatal conductance; the higher the stomatal conductance, the higher will be the CO_2_ absorption and energy production. However, salt stress decreases CO_2_ fixation; as a result, reactive oxygen species (ROS) is produced by the leakage of electron into O_2_ (Ahammed et al., 2018). Cotton growth and plant development comprising plant height, fresh and dry weights, plant weight, root to shoot ratio, leaf area and canopy development, and other physiological parameters like photosynthesis (Pn), transpiration rate (Tr), stomatal conductance (St), overall yield, and primarily fiber quality were severely affected by salinity (Loka et al., 2011). These detrimental effects of salt (NaCl) on cotton differ in accordance with the change in salt concentration (i.e., 10, 20 dS/m, etc.), depending upon the time period of salt exposure (how long it will be) and growth stage in which the plant is exposed to stress (i.e., germination or emergence). Cotton is referred as a salt-tolerant crop after barley, but its yield falls by 5% per unit dS/m with the increase in stress limit (Chinnusamy et al., 2005). Thus, the 200 mM NaCl (20 dS/m) stress treatment would induce an approximately 60% yield reduction underneath the field conditions (Higbie et al., 2010).

Salt stress induced many physiological and biochemical impairments in plants such as photosynthesis, ionic imbalance, and oxidative injury to proteins (enzymes) (Zhu, 2001; Xiong and Zhu, 2002; Muchate et al., 2016). However, the main factor under salt stress condition in cotton is the positive ion (Na^+^) instead of (Cl^–^) (Gouia et al., 1994). Under salinity stress, the Na^+^ content in the shoots (cotyledon, leaves, and stems) is greater than that in the roots. In leaves, different types of cells have different capacity of sodium accumulation, which is more in the epidermal cell rather than in the mesophyll cell, and this was mainly noted in sensitive genotypes (Peng et al., 2016). Similarly, the salt-tolerant genotypes have aptitude of maintaining K^+^/Na^+^ ratios. For osmotic adjustment, the Na^+^ sequestration into the vacuole is very important in order to minimize the Na^+^ concentration in cell cytoplasm (Maathuis, 2013). For this purpose, Na^+^/H^+^ antiporters (Apse et al., 1999; Shi and Zhu, 2002) V-ATPase and V-PPase [vacuolar (V)], two H^+^ pumps, are involved in Na^+^ compartmentalization into the vacuoles (Dietz et al., 1969). Similarly, the salt-tolerant genotype has more capability of Na^+^ repossession into the vacuoles. High concentration of NaCl mainly causes ion toxicity, osmotic stress, and mineral disruption (such as those of K^+^ and Ca^2+^) in plants (Zhu, 2003; Munns and Tester, 2008).

Salt stress also affects the metabolic activities of plant tissues by overproduction of reactive oxygen species (such as O^2–^, ^1^O_2_, and ^∙^OH) in the cell, which oxidize different biochemical compounds like proteins, lipids, DNA, and RNA and enters the plant into oxidative stress (Munns and Tester, 2008; Miller et al., 2010). Plants possess various extensive antioxidative mechanisms in order to regulate the reactive oxygen species, for instance, ascorbate peroxidase (APX) pathway and superoxide dismutase (SOD) pathway for protection against reactive oxygen destruction. SOD pathway is a first course of action against ROS. It is employed as ROS scavenger to cope with salt stress by converting O^2–^ into H_2_O_2_ and O_2_ in roots (Alscher et al., 2002). Similarly H_2_O_2_ could get H_2_O and O_2_ by reduction reaction in catalase (CAT) pathway confined in peroxisomes organelle (Miller et al., 2010). Peroxidase (POD) is a heme-containing glycoprotein, which catalyzes the H_2_O_2_ in reduction reaction by using different electron donors for example phenolic compounds and secondary metabolites (Hiraga et al., 2001; Zhao et al., 2013). The superoxide dismutase (SOD) activity is enhanced under salt stress. Likewise, malondialdehyde (MDA) content is increased with the rising in lipoxygenase (LOX) activity, as LOX act as a catalyst in the production of fatty acid. Antioxidant enzymes activity like POD and catalase (CAT) is lower in salt-sensitive cultivars than in the tolerant cotton genotypes. In addition, cotton genotypes that are tolerant to salinity exhibit greater activities of SOD, ascorbate, POD, and glutathione reductase with less CAT activity as the amount of salt increases (Jabran et al., 2019). In cotton, the expression of SOD enhanced with respect to the rise in NaCl concentration, whereas POD activity is up to 53% higher in tolerant cultivars. Higher POD activity has improved photosynthesis, which shows the part of antioxidants defense system to alleviate salinity stress (Zhang and Lin, 2014; Sharif et al., 2019). Ascorbate/ascorbic acid is another essential non-enzymatic antioxidant that is higher in the cytoplasm and cell chloroplast when exposed to salt stress. Ascorbic acid has the ability to maintain the photosynthetic apparatus in chloroplast under salinity. In many genotypes, more ascorbic acid in their cell at early growth stages seems to be an indication of tolerance (Aslam et al., 2013).

The present study is designed to screen salt-tolerant genotypes at their seedling stage. As the seedling stage is the most critical stage in proper development of plant systems and processes, therefore, it is important to examine the changes and modifications that arise due to salt stress. The information obtained from this work would be use to identify salt-tolerant and salt-susceptible genotypes and the proper utilization of saline and arid land in order to expand cotton cultivation area. Moreover, this study also provides effective breeding strategies to enhance salt tolerance in cotton.

## Materials and Methods

### Collection of Cotton Germplasm

In order to conduct the present study, healthy seeds of 22 genotypes were collected from the Central Cotton Research Institute (CCRI) Multan and Nuclear Institute for Agriculture and Biology (NIAB) Faisalabad (Table 1).

**TABLE 1:** List of genotypes used in first experiment (Petri plate).

**Sr. No.**	**Genotypes**	**Sr. No.**	**Genotypes**
(1)	ROHI 1	(12)	CIM-779
(2)	SASUI-2018	(13)	6071/16
(3)	SITARA-16	(14)	FH-326
(4)	MNH-1026	(15)	FH-152
(5)	MNH-1020	(16)	IR-NIBGE-13
(6)	CMB-CLEAN COTTON-1	(17)	GH-HADI
(7)	CRIS-671	(18)	BS-2018
(8)	WEAL-ag-08	(19)	VH-189
(9)	IUB-69	(20)	RH-670
(10)	BT-CIM-678	(21)	NIAB-512
(11)	CIM-602	(22)	NIAB-135

### Initial Screening Using Germination Test in Petri Plates

First of all, the delinted seeds were imbibed in 15 mM aerated CaSO_4_.2H_2_O for 3 h and then treated with fungicide 1% Topsin M for 2–3 min. After washing the treated seeds with distilled water three times, they were placed on the germination paper (filter) in Petri plates that had been moistened with 0, 7.5, and 15 dS/m NaCl solutions. Each plate contained 10 seeds arranged in equal distance, and plates were properly labeled according to genotype as well as treatment. The Petri plates were placed in a growth chamber at running 29/19°C (day/night) temperature with light intensity of 550 μm m^–2^ s^–1^ 16/8 h (light/dark) photoperiod. After 4 days, the germination data were recorded. If the emerging radical of the seed was longer than of seed length or the length of radical taken was ≥ 0.5 cm, then it was considered as germinated seed. The Petri plates were kept for 10 days in a growth chamber, and germination data were recorded regularly for analysis. The relative germination rate was calculated as follows:

RGR = (no. of germinated seed in salt stress condition/no. of germinated seed in control condition) × 100%

### Germination Parameters

Final germination (FG), mean germination time (MGT), and germination index (GI) were estimated using the following formulae:

FG=(finalno.ofgerminatedseed/no.ofseedsinPetriplate)×100

MGT=(n1×t1+t2×n2+t3×n3…)/⁢(n1+n2+n3+n4⁢…)

where n = no. of germinated seed.

t = germination time interval.

G⁢I=n⁢1/d⁢1+n⁢2/d⁢2+n⁢3/d⁢3⁢…

where n = no. of germinated seed.

d = 1st, 2nd, and 3rd day, respectively.

### Screening of Salinity Tolerance at Seedling Stage Under Glasshouse Conditions

Eleven genotypes were selected (including sensitive and tolerant) on the basis of germination parameters, i.e., MGT and GI, etc. through the initial screening of Petri plates experiment. These genotypes were further screened under control and saline conditions with increased salt concentration (Table 2).

**TABLE 2:** Selected genotypes for seedling experiment under glass house conditions.

**Sr. No.**	**Genotypes**	**Sr. No.**	**Genotypes**
(1)	SITARA-16	(7)	IR-NIBGE-13
(2)	CIM-602	(8)	GH-HADI
(3)	CIM-779	(9)	BS-2018
(4)	6071/16	(10)	NIAB-512
(5)	FH-326	(11)	NIAB-135
(6)	FH-152		

**FIGURE 1:**
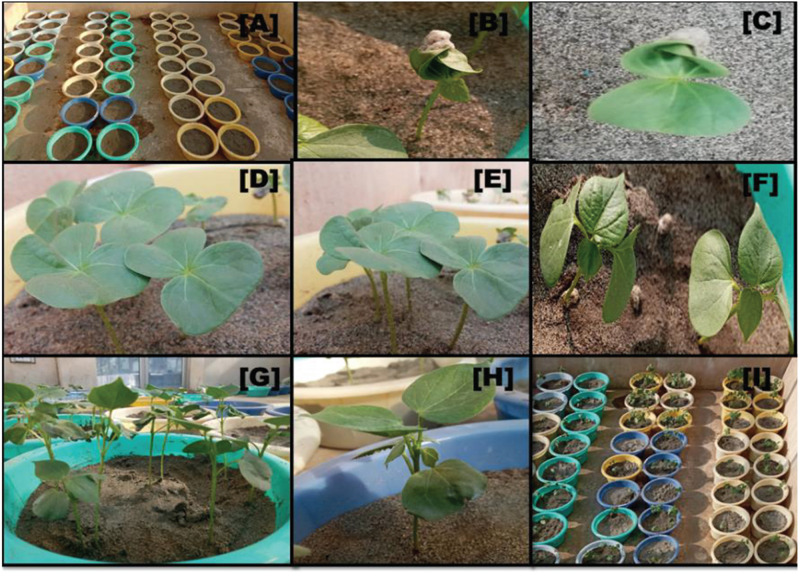
Visual appearance of seedling emergence under glass house conditions: **(A)** sowing of seeds in sand pots; **(B,C)** epicotyl emergence of cotyledon leaf 4 DAT sowing; **(D,E)** full emergence of cotyledon leaves; **(F)** first and second true leaves emergence; **(G,H)** seedlings at fourth true leave stage; and **(I)** the overall view of sand pots before the day of harvesting.

Seeds were planted during November 2019 in circular pots (top width, 27 cm; bottom width, 20.5 cm) that were filled with 4 kg sand. All the pots were watered at field capacity before sowing. For sowing, linted seeds were soaked in 15 mM aerated CaSO_4_.2H_2_O overnight. In the next morning, 8–10 seeds were sown approximately 2 cm deep in a sand pot. Every genotype with three replicates and treatments of 0, 15, and 20 dS/m of NaCl was maintained in completely randomized design. Glasshouse temperature was kept at ∼35°C daytime and ∼20°C nighttime using electric bulbs with a light intensity at 2500 lx for 14 h. After 3–4 days of sowing, the pots were watered with Hoagland solution of one-eighth strength with field capacity of sand, and this strength was subsequently increased to one-fourth when the cotyledon leaves emerged (Figure 1). When the first true leaves appeared after 15 days of sowing, they were maintained under one-half strength of NaCl-free Hoagland solution until the second true leaf appeared. At the early emergence of the third true leaf, the first salt stress of 50 mM was given, which was increased afterward and maintained one set under 15 dS/m (150 mM) and another one at 20 dS/m (200 mM) of NaCl; however, no NaCl was added to the set that served as control, respectively. After treatment, different physiological traits were recorded between 11 AM and 2 PM on the day before harvesting. Afterward, the plants along with the roots were harvested, and then, different morphological traits were measured.

### Morphological Parameters

At three true leaves stage, the following morphological data were recorded for analysis: The plants of each genotype were rinse with dH_2_O thoroughly, and then, the root and shoot lengths were measured in centimeters. The mean values for each genotype were calculated in each treatment for further analysis. The weight of the whole plant along with roots, shoots, and leaves was measured with the help of weighing balance. After recording the fresh weight, plants were kept in drying oven at 70°C for 48 h. Then, dry weights of plants were estimated on an electrical weighing balance.

### Physiological Parameters

Different physiological traits like photosynthesis, stomatal conductance, transpiration, and chlorophyll content were measured by using LI-1600 Steady State Porometer and chlorophyll content by using SPAD-502; Na^+^/K^+^ ratio was also measured.

Stomatal conductance (mmol m^–2^ s^–1^) of control and treated plants was recorded with the help of a porometer and then calculated by using the formula:

Stomatal conductance (Sc) = 1/Dr × CF

Dr = diffusible resistance, which was calculated from the porometer;
CF = correction factor, which was calculated using formula: LT × constant.

The constant value at 25°C for durum was 411.8, and leaf temperature (LT) was also measured by the porometer. In the same manner, stomatal conductance of each control and treatment plants was calculated. Photosynthetic rate (μmol m^–2^ s^–1^) was calculated from transpiration rate and stomatal conductance by using the formula:

Phr = Sc (mmol m^–2^ s^–1^)/Tr (μg cm^–2^ s^–1^) × 10

Sc = stomatal conductance; Tr = transpiration rate.

Transpiration rate (mmol m^–2^ s^–1^) was measured in μg cm^–2^ s^–1^, as relative value was calculated by the transpiration value from the porometer divided by 10,000 and multiplied with 1000. A chlorophyll meter (Model: SPAD 502 plus Japan) was used to measure the chlorophyll contents in the leaves before and after salt stress. The chlorophyll meter SPAD was placed on the uppermost leaf of a plant to measure the chlorophyll. The day before harvesting, physiological data were recorded for analysis under control and saline conditions.

#### Na^+^/K^+^ Ratio

Sodium (Na^+^) and potassium (K^+^) concentrations were measured on a flame photometer (Model; Jenway PFP 7). First of all, to digest dried ground material, 0.05g of the leaf material was taken in digestion tubes. Then, 1 ml of conc.H_2_SO_4_ was added on the dried sample for the sake of digestion. All the tubes were kept in the dark for overnight incubation at room temperature. The next day, 0.5 ml of H_2_O_2_ (35%) was added, and the tubes were shifted in a digestion block and heated at 350°C up to the production of fumes. After 30 min of heating, the digestion tubes were removed from the block to cool down. H_2_O_2_ (0.5 ml) was added gradually, and the tubes were positioned back into the digestion block. The above mentioned step was repeated until the color of the digestion material turned transparent. Then, the volume of the digestion extract made up 25 ml of the volumetric flasks. After the extract was filtered, it used to analyze K^+^ and Na^+^.

### Stress Susceptibility Index

Stress susceptibility index (SSI) was calculated for the tested genotypes under 15 and 20 dS/m salt stress and non-stress conditions by using this formula (Fischer and Maurer, 1978):

SSI=1-(Ys/Yp)/SI

Ys = means of characters under salt stress conditions;
Yp = mean of character under non-stress condition.

While for stress intensity (SI):

SI = 1−(Ŷs/Ŷp)

Ŷs = means of all the genotypes under stress conditions.
Ŷp = means of all the genotypes under non-stress condition.

### Stress Tolerance Index

Stress tolerance index (STI) was calculated for the determination of stress tolerance potential of genotypes under 15 and 20 dS/m salt stress and non-stress conditions by using following formulae (Fernandez, 1993):

Root length STI = (root length of stressed plant/root length of non-stressed plant) × 100
Shoot length STI = (shoot length of stressed plant/shoot length of non-stressed plant) × 100
Stomatal conductance STI = (stomatal conductance of stressed plant/stomatal conductance of non-stressed plant) × 100
Photosynthetic rate STI = (photosynthetic rate of stressed plant/photosynthetic rate of non-stressed plant) × 100
Transpiration rate STI = (transpiration rate of stressed plant/transpiration rate of non-stressed plant) × 100
Chlorophyll content STI = (chlorophyll content of stressed plant/vhlorophyll content of non-stressed plant) × 100

### Biochemical Analysis

After morphophysiological analysis, treatment-induced biochemical modifications in plant leaves were analyzed. The genotypes were selected on the basis of variance analysis of morphophysiological parameters, SSI and STI; the two sensitive and five tolerant genotypes were selected and further tested by biochemical analysis (Table 3).

**TABLE 3:** Selective sensitive and tolerant genotypes for biochemical analysis.

**Sr. No.**	**Genotypes**	**Sr. No.**	**Genotypes**
(1)	6071/17	(5)	IR-NIBGE-13
(2)	FH-326	(6)	GH-HADI
(3)	FH-512	(7)	NIAB-512
(4)	NIAB-135		

### Extraction for Antioxidant Enzyme Activities

First of all, after weighing, 0.1 g fresh leaf from all cotton genotypes was extracted in 1 ml of 50 mM potassium phosphate buffer with pH 7.4, and then, ground samples were centrifuged at 14,000 × *g* for 10 min at 4°C. The resultant supernatant was separated and utilized for determination of different enzymatic and non-enzymatic analysis. All the data were taken in duplicate.

#### Enzymatic Antioxidants

##### Ascorbate oxidase activity

For estimation of APX activity, leaf sample was homogenized in 50 mM potassium phosphate buffer, and APX was measured using the method explained by Dixit et al. (2001). The required reagent R1 assay buffer was prepared by using 0.2 M potassium phosphate buffer (pH 7.0), 10 mM of ascorbic acid, and then 20 ml of 0.5 M ethylenediaminetetraacetic acid (EDTA) mixing to get assay buffer. The second reagent R2 was 4 mM H_2_O_2_, and then the sample was extracted. For the estimation of APX activity, 1 ml assay buffer and 50 μl sample were added, and the reaction was initiated after adding 1 ml of 10% H_2_O_2_. The decrease in oxidation rate of ascorbic acid in absorbance at 290 nm after every 30 s was measured.

##### Superoxide dismutase activity

First, cotton leaves were ground in 50 mM potassium phosphate buffer (pH 7.0), 0.1 mM EDTA, and 1 mM dithiothreitol (DTT) as described in Dixit et al. (2001). For estimation of SOD activity, the samples after adding reagents [400 μl water, 250 μl of 0.2 M potassium phosphate buffer, 100 μl L-methane, 100 μl Triton X, 100 μl nitroblue tetrazolium (NBT)] were kept under the white light for 10 min. SOD activity was analyzed by assessing its ability to prevent the photochemical reduction in NBT that is present in chemical reagents, as explained in the method in Giaanopolitis and Ries (1977). Thus, 1 U of SOD activity is explained by the amount that produced 50% resist photochemical reduction in NBT.

##### Peroxidase activity

In order to analyze the POD activity, cotton leaves were homogenized in 50 mM potassium phosphate buffer having pH 7.0 and POD activity estimated by using this method (Maehly and Chance, 1955) with certain amendments. Thus, as to POD measurement, the assay solution prepared by mixing distilled water (535 μl), 200 mM phosphate buffer with pH 7.0, 200 mM guaiacol, 400 mM H_2_O_2_, and 15 μl extracted sample. After adding the enzyme extract, reaction was began and absorbance at 470 nm was recorded after every 20 s. The increasing trend in absorbance was observed, and enzyme activity was explained on the basis of leaves weight. Thus, 1 U of POD activity was defined as an absorbance change of 0.01/min.

##### Catalase activity

For the estimation of catalase activity, cotton leaves were emulsified in 50 mM potassium phosphate buffer (pH 7.0) and 1 mM dithiothreitol (DTT), and estimation was done by the method explained by Beers and Sizer (1952). The required assay solution for CAT activity contained 50 mM potassium phosphate buffer with same pH 7.0, 59 mM H_2_O_2_, and 0.1 ml sample (enzyme extract). The reaction was started after adding the sample and decreasing pattern in absorbance at 240 nm was recorded after every 20 s. Thus, 1 U of CAT activity described as change in absorbance of 0.01/min and enzyme activity expressed on the basis of leaves weight.

##### Total antioxidant capacity

The reagent 1 (R1), 400 mmol/l acetate buffer solution (pH 5.8), was prepared by suspending 54.432 g of CH_3_COONa.3H_2_O in 1 L deionized water. A 60-ml acetic acid was mixed with sodium acetate solution. The reagent 2 (R2), 30 mmol/l acetate buffer solution (pH 3.6), was prepared by adding 2.46 g CH_3_COONa suspended in 1 L of deionized water. Acetic acid after dilution was mixed with sodium acetate solution. Then, 278 μl was removed from R2, and the same amount was added from 35% of H_2_O_2_ solution. 2,2′-Azinobis-3-ethylbenzthiazolin-6-sulfonic acid (ABTS) (0.549 g) was suspended in 100 ml of already equipped solution (final concentration, 10 mmol/L). The characteristic color of ABTS appeared after incubation for 1 h at room temperature. Colored reagent is unstable for 6 months if stored at 4°C. The first absorbance of this assay was taken before mixing R1 and R2, which served as blank, and the next absorbance was measured after mixing R1 and R2 (after 5 min incubation at room temperature).

#### Non-enzymatic Antioxidants

##### Total phenolic content

A total phenolic content (TPC) estimation method as explained in Ainsworth and Gillespie (2007) was applied with certain modifications in order to estimate TPC. First of all, 0.05 g of leaves sample was weighed, and for homogenization, it was placed in the dark for 48 h after adding 500 μl chilled 95% methanol and then centrifuged at 14,000 × *g* for 5 min. The supernatant was separated and used for TPC estimation. The assay protocol was as follows: 150 μl of 10% (v/v) F-C reagent mixed with 100 μl sample vortex thoroughly and then added 1.2 ml of 700 mM Na_2_CO_3_. Then, samples were left for 1 h incubation at room temperature. Blank corrected absorbance of samples was recorded at 765 nm. Phenolic content (gallic acid equivalents) of samples was determined by using linear regression equation.

##### Tannins

The supernatant from TPC assay was not discarded after recording readings from a spectrophotometer. PVP.P (0.1 g) was added in TPC samples and vortexed for 2–3 min. Then, these samples were centrifuged at 14,000 × *g*, and the supernatant was used for absorbance at 765 nm so as to estimate tannins in cotton leaves sample.

##### Ascorbic acid

The 2,6-dichloroindophenol (DCIP) method was used for ascorbic acid determination as explained by Hameed et al. (2005). For concise description, each molecule of vitamin C converted DCIP molecule into DCIPH_2_ molecule, and this reaction was examined as a decreasing trend in absorbance at 520 nm was observed. A standard curve and linear regression equation was used to find the ascorbate concentration in the samples.

##### Alpha amylase activity

The alpha amylase activity of the cotton leaves was determined by the method in Varavinit et al. (2002) with certain modifications. Two reagents were required for α-amylase estimation. One is 3,5-dinitrosalicyclic acid (DNS) (prepared with 1 g DNS in dH_2_O, then added 30 g of sodium potassium tatrate tetrahydride, and 20 ml of 2 N NaOH made up volume up to 100 ml), and the second was 1% starch solution. After adding 0.2 ml sample and 1% starch solution, the reaction mixture was incubated for 3 min and placed in water bath for 15 min after adding DNS, and then made up volume up to 9 ml with dH_2_O. Absorption was observed at 540 nm spectrometrically.

##### Reducing sugars (sugar content)

The reducing sugar level in cotton leaves was estimated by using dinitrosalicyclic acid method explained in Miller (1959). Thus, the total soluble sugar contents of cotton leaves were also measured by phenol-sulfuric acid reagent method (Dubois et al., 1956). In this way, the non-reducing sugars were determined by the difference in reducing and total soluble sugars.

#### Other Biochemical Parameters

##### Pigment analysis

The amount of chlorophyll (a and b) and carotenoids was calculated by using method described in Arnon (1949). In the first step, 0.075 g of cotton leaves samples was homogenized in 80% chilled acetone and incubated at room temperature for 24 h in dark. After 24 h, it was centrifuged at 14,000 × *g* for 5 min. Absorbance of supernatant was measured at 645, 663, 505, 453, and 470 nm.

##### Total oxidant status

Total oxidant status (TOS) in cotton leaves samples was determined by the method described in Erel (2005). This method is based on the oxidation of Fe^+2^ ions into Fe^+3^ by oxidants that are present in the sample acidic medium and measurement of ferric ions by xylenol orange. The assay mixture contained reagent R1, which is stock xylene orange solution containing 0.38 g in 500 μl of 25 mM H_2_SO_4_, 0.4 g of NaCl, 500 μl of glycerol, and volume up to 50 ml with 25 mM H_2_SO_4_. The reagent R2 contained 0.0317 g of o-dianisidine and 0.0196 g of ferrous ammonium sulfate and sample extract. After 5 min of adding R2, the absorption was measured at 560 nm by using a spectrophotometer. A standard curve was prepared using hydrogen peroxide. The results were expressed in μM H_2_O_2_ equivalent per L.

##### Total soluble proteins

To determine the protein content, leaves samples were homogenized in a medium of potassium phosphate buffer. Quantitative protein determination was done by using the method Bradford (1976). The assay protocol contained 5 μl of supernatant of the sample extract, 0.1 N NaCl, and then mixed with 1.0 ml of Bradford dye. This mixture was kept for 5 min to form protein dye complex before taking readings. Blank-corrected reading was calculated at 595 nm by a spectrophotometer.

##### Malondialdehyde content

The lipid peroxidation level in cotton leaves was assessed regarding MDA (a product from lipid peroxidation) content measured by thiobarbituric acid (TBA) method described in Heath and Packer (1968) with slight modification of the method in Dhindsa et al. (1981). The 0.1-g leaves sample was homogenized in 0.1% TCA. This extract was centrifuged at 14,000 × *g* for 5 min. To 1 ml aliquot of the supernatant, 20% TCA containing 0.05% TBA was added. The mixture was heated at 95°C for 30 min and then quickly cooled in an ice bath. After centrifuging at 14,000 × *g* for 10 min, the absorbance of the supernatant was recorded at 532 nm, and the value for the non-specific absorption at 600 nm was subtracted. The MDA content was estimated by using extinction coefficient of 155 mM^–1^ cm^–1^.

##### Total flavonoids

The total flavonoid (TF) content was determined by using aluminum chloride colorimetric method (Ainsworth and Gillespie, 2007). Each sample extract was mixed with 0.1 ml of 10% aluminum chloride hexadihydrate, 0.1 ml of 1 M potassium acetate and 2.8 ml of deionized water. After 40 min of incubation at the room temperature, the absorbance of the sample was determined by a spectrophotometer at 415 nm.

#### Statistical Analysis

Data were presented in the graphs as mean values and standard error. Statistical analysis was based on variance analysis and Tukey [honestly significant difference (HSD)] test at p < 0.05. The different letters above the bars in the same genotypes under stress and non-stress conditions indicated significant differences with tolerance 0.0001. Bars with similar letters in the same genotypes under treatments were non-significant. Principal component analysis (PCA) and correlation (Pearson test) were performed by using XL-STAT 2012.1.02 with 95% confidence interval. Cluster analysis was also performed by agglomerative hierarchical clustering (AHC) of cotton genotypes under control and salt stress conditions.

## Results

### Seed Germination Test

**FIGURE 2:**
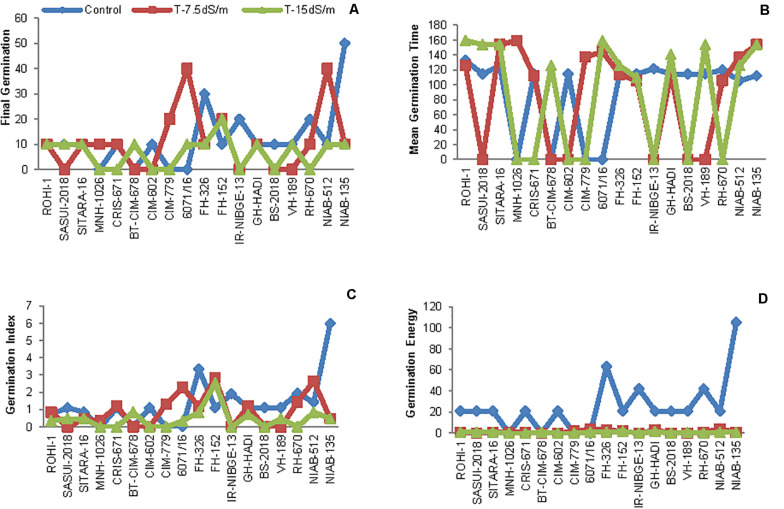
**(A)** Final seed germination of cotton, **(B)** mean germination time (MGT), **(C)** germination index (GI), and **(D)** germination energy (GE) in growth chamber under control and salt stress conditions.

**FIGURE 3:**
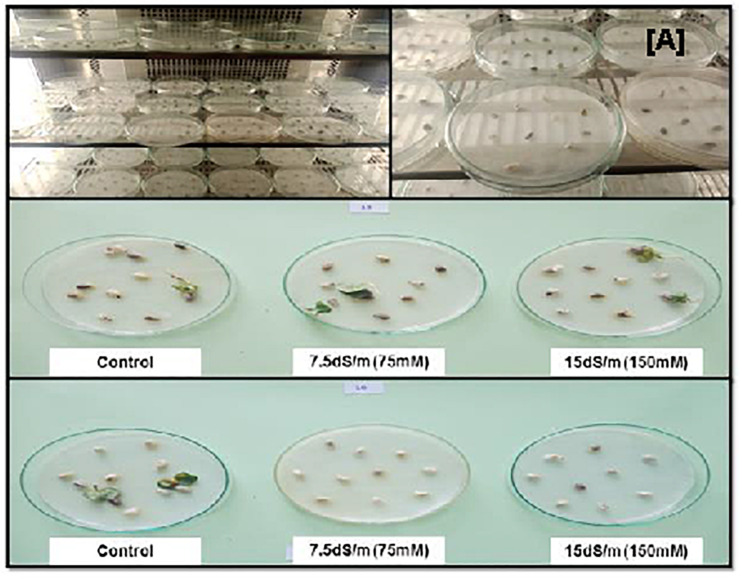
**(A)** Cotton seeds germination in growth chamber under salinity stress and normal condition. The tolerant cotton seed germination occurs in both salt stresses but no germination in sensitive genotypes.

The initial screening was done at seed germination stage. In this experiment, seeds were tested against 0, 7.5, and 15 dS/m salt stress. Genotypes with 0% germination even in control, i.e., MNH-1020, CMB-CLEAN-COTTON, WEAL-AG-08, and IUB-69, were excluded. Salt stress inhibited seed germination in many genotypes. In NIAB-512, seed germination percentage remained the same as in control and under 15 dS/m, but 30% germination was increased under 7.5 dS/m salt stress condition. Similarly, 6071/16 also showed increase in 30% germination under 7.5 dS/m, and both genotypes behaved as moderately tolerant. In IR-NIBGE-13, seed germination occurred in control but not under stress conditions and was regarded as sensitive genotype at germination stage. The seed germination was slightly attenuated by salt stress in NIAB-135, as only 20% germination occurred. Moreover, in FH-152, germination was increased by 20% under stress conditions (Figures 2A, 3).

The relative increase in mean germination time (MGT) was observed in ROHI-1, SITARA-16, FH-326, 6071/16NIAB-135, and NIAB-512 under stress conditions as compared to that under non-stress condition. Salt stress showed no significant effect in FH-152, as MGT of seeds remained the same under control and stress conditions (Figure 2B).

Salt stress significantly decreased germination index (GI) in FH-326 and NIAB-135. The maximum GI was recorded in NIAB-135, afterward in FH-326, under non-stress condition. In contrast, GI was increased in FH-152 under stress conditions. However, the least differences between control and stress were observed in ROHI-1, SITARA-16, and NIAB-512 (Figure 2C).

In addition, seed germination energy (GE) was highly retarded by salt stress. An increased in GE was observed more under non-stress as compared to salt stress conditions in all the genotypes. The maximum GE was recorded in NIAB-135 and then in FH-326 under non-stress condition (Figure 2D).

### Morphological Parameters

#### Root and Shoot Lengths

**FIGURE 4:**
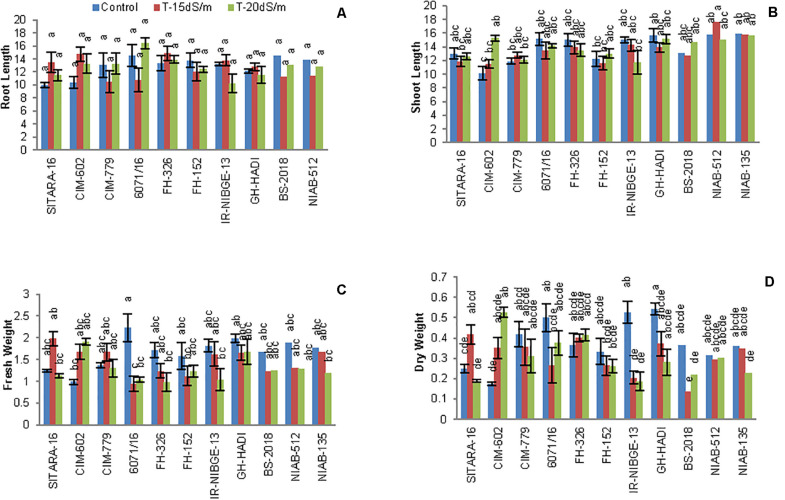
Comparison of panels **(A)** root length, **(B)** shoot length, **(C)** fresh weight, and **(D)** dry weight between cotton genotypes under control and salt stress conditions.

**FIGURE 5:**
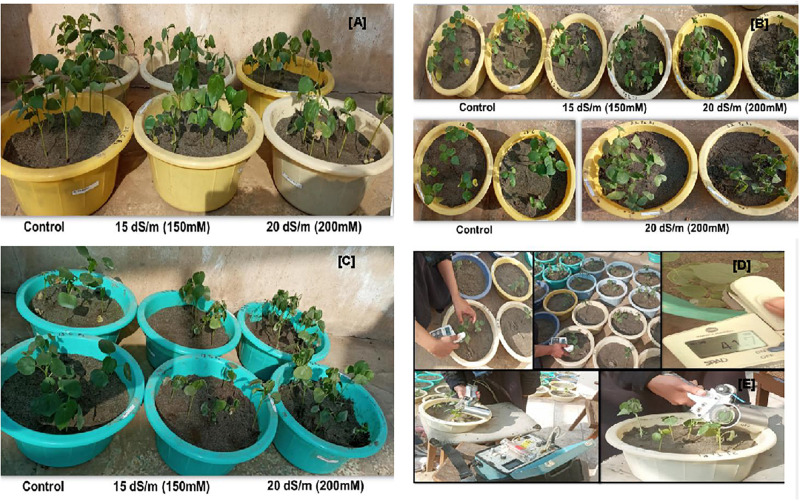
Pictorial view of panels **(A)** NIAB-512, **(B)** NIAB-135, and **(C)** 6071/16 under control and salt stress conditions (sand pot in glass house) at fourth true leave stage. Schematic diagram of recording physiological parameters: Measuring chlorophyll content by using **(D)** SPAD and stomatal conductance, transpiration, and photosynthetic rate before harvesting by using **(E)** porometer.

The growth parameters root and shoot lengths were not significantly affected by NaCl treatment, with the exception of CIM-602 and NIAB-512 in which the shoot length was significantly increased compared to that in non-stress condition. However, most of the genotypes had not shown considerable changes in root and shoot lengths (Figures 4A,B, 5).

On the basis of STI, CIM-602, GH-HADI, and FH-326 found to be tolerant genotypes for root length under stress conditions. All the accessions were also cross-checked with SSI. Under 20 dS/m salt stress conditions, IR-NIBGE-13 and FH-152 were found to be susceptible for root length (Table 4). Similarly, CIM-602, NIAB-512, NIAB-135, CIM-779, and FH-326 showed more STI values for shoot length under stress conditions.

**TABLE 4:** Stress susceptibility and tolerance indices for shoot and root lengths.

**Genotypes**	**Shoot length**				**Root length**			
	**SSI-T1**	**SSI-T2**	**STI-T1**	**STI-T2**	**SSI-T1**	**SSI-T2**	**STI-T1**	**STI-T2**
SITARA-16	3.832776	−176.308	0.913462	0.971154	−9.18668	−22.7716	1.35	1.15
CIM-602	−6.01467	3093.728	1.135802	1.506173	−11.0683	−42.0681	1.421687	1.277108
CIM-779	−3.26346	128.6737	1.073684	1.021053	5.047628	−2.91944	0.807692	1.019231
6071/16	4.758414	−404.099	0.892562	0.933884	6.788189	−19.6307	0.741379	1.12931
FH-326	3.321739	−611.2	0.925	0.9	−2.94366	−5.67517	1.11215	1.037383
FH-152	2.259686	374.2041	0.94898	1.061224	3.340612	15.18108	0.872727	0.9
IR-NIBGE-13	2.214493	−1324.27	0.95	0.783333	−0.99048	34.37226	1.037736	0.773585
GH-HADI	4.960464	−195.584	0.888	0.968	−1.35297	7.8253	1.051546	0.948454
BS-2018	1.265424	698.5143	0.971429	1.114286	5.883097	14.39585	0.775862	0.905172
NIAB-512	−5.2726	−291.048	1.119048	0.952381	4.729309	12.30898	0.81982	0.918919
NIAB-135	0.556231	−86.3548	0.987441	0.985871	4.721207	5.526304	0.820128	0.963597

*SSI-T1, stress susceptibility index under 15 dS/m salt stress; SSI-T2, stress susceptibility index under 20 dS/m salt stress; STI-T1, stress tolerance index under 15 dS/m salt stress; STI-T2, stress tolerance index under 20 dS/m salt stress.*

#### Plant Fresh and Dry Weights

In cotton plants, NaCl treatment significantly reduced plant biomass in two genotypes IR-NIBGE-13 and BS-2018. A similar trend was also noted in other genotypes. However, NIAB-135, NIAB-512, and GH-HADI had the least difference in fresh weight between non-NaCl and NaCl-treated plants (Figures 4C,D).

Plant fresh weight was significantly reduced in 6071/16. Similarly, on the basis of SSI and STI, 6071/16 found to be susceptible for fresh weight and IR-NIBGE-13, 6071/16, and BS-2018 for dry weight under stress conditions. The fresh and dry weights were least affected by salt stress in NIAB-512 and NIAB-135, with the exception of NIAB-135, which showed susceptibility under 20 dS/m salt stress for dry weight. Although salt stress had a significant effect on plant biomass, FH-152, FH-326, NIAB-512, and NIAB-135 improved significantly by maintaining their dry weights compared to that under control (Table 5).

**TABLE 5:** Stress susceptibility and tolerance Indices for fresh and dry weights.

**Genotypes**	**Fresh weight**				**Dry weight**			
	**SSI-T1**	**SSI-T2**	**STI-T1**	**STI-T2**	**SSI-T1**	**SSI-T2**	**STI-T1**	**STI-T2**
SITARA-16	−4.94974	0.40731	1.589537	0.905433	−3.7946	1.189054	1.68008	0.754527
CIM-602	−5.8453	−4.03449	1.696203	1.936709	−5.61152	−9.75014	2.005714	3.012857
CIM-779	−1.8046	0.219669	1.214936	0.948998	0.850737	1.243439	0.847528	0.7433
6071/16	4.872994	2.297404	0.419604	0.4666	2.625384	1.209773	0.529471	0.75025
FH-326	2.346475	1.849478	0.720524	0.570597	−0.51797	−0.63153	1.092833	1.130375
FH-152	2.453801	0.954636	0.707741	0.778357	1.156141	1.036427	0.792793	0.786036
IR-NIBGE-13	0.889258	1.836586	0.894085	0.57359	3.42651	3.13296	0.38589	0.35322
GH-HADI	1.402846	0.665543	0.832915	0.845477	1.76483	2.34391	0.683702	0.516114
BS-2018	2.20583	1.108366	0.737275	0.742665	3.461762	1.908824	0.379572	0.605935
NIAB-512	2.575659	1.372777	0.693227	0.681275	0.403614	0.161721	0.927663	0.966614
NIAB-135	0.523862	1.425586	0.937606	0.669014	0.170252	1.807237	0.969487	0.626907

*SSI-T1, stress susceptibility index under 15 dS/m salt stress; SSI-T2, stress susceptibility index under 20 dS/m salt stress; STI-T1, stress tolerance index under 15 dS/m salt stress; STI-T2, stress tolerance index under 20 dS/m salt stress.*

### Physiological Parameters

#### Stomatal Conductance and Photosynthetic Rate

**FIGURE 6:**
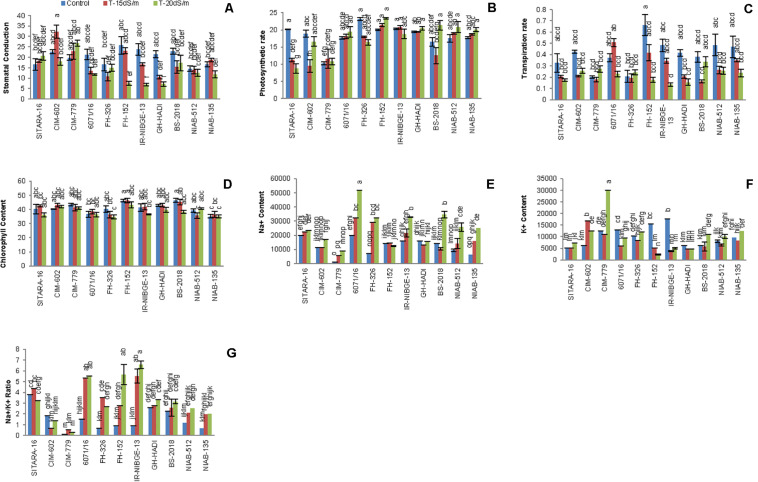
Comparison of panels **(A)** stomatal conductance, **(B)** photosynthetic rate, **(C)** transpiration rate, **(D)** chlorophyll content, **(E,F)** Na^+^ and K^+^ content, and **(G)** Na + /K + ratio between the cotton genotypes under normal and salt stress conditions.

Salt stress significantly decreased stomatal conductance in IR-NIBGE-13, 6071/16, GH-HADI, and FH-152 (Figure 6). However, stomatal conductance was maintained in NIAB-135, NIAB-512, SITARA-16, and FH-326 under stress and non-stress conditions (Table 6). Photosynthetic rate was maintained in all the genotypes with the exception of SITARA-16 and CIM-602. Based on SSI, IR-NIBGE-13 was found to be more sensitive under 20 dS/m salt stress condition (Table 7).

**TABLE 6:** Stress susceptibility and tolerance indices for stomatal conductance and transpiration rate.

**Genotypes**	**Stomatal conductance**				**Transpiration rate**			
	**SSI-T1**	**SSI-T2**	**STI-T1**	**STI-T2**	**SSI-T1**	**SSI-T2**	**STI-T1**	**STI-T2**
SITARA-16	−0.87014	−0.7958	1.100472	1.239213	19.52062	0.938179	0.6298	0.529954
CIM-602	−3.72031	0.687488	1.42957	0.793345	26.67516	0.786631	0.494118	0.605882
CIM-779	−1.34662	−1.16711	1.155489	1.350826	5.858886	−0.68995	0.888889	1.345679
6071/16	3.137208	1.498992	0.637758	0.549411	−19.1102	0.776939	1.362416	0.610738
FH-326	3.029496	0.321093	0.650196	0.903481	3.451417	−0.3508	0.934545	1.175758
FH-152	0.860658	2.357692	0.900623	0.29129	19.55694	1.467706	0.629112	0.26465
IR-NIBGE-13	2.628489	2.36348	0.696498	0.28955	15.13596	1.440585	0.712953	0.278238
GH-HADI	4.404499	2.215894	0.491429	0.333914	26.68264	1.251664	0.493976	0.372892
BS-2018	2.826926	0.814131	0.673586	0.755277	30.18097	0.223229	0.427632	0.888158
NIAB-512	0.367756	0.458233	0.957537	0.862257	24.19056	0.936235	0.541237	0.530928
NIAB-135	−1.12746	0.94688	1.130184	0.715373	13.25299	0.981954	0.748663	0.508021

*SSI-T1, stress susceptibility index under 15 dS/m salt stress; SSI-T2, stress susceptibility index under 20 dS/m salt stress; STI-T1, stress tolerance index under 15 dS/m salt stress; STI-T2, stress tolerance index under 20 dS/m salt stress.*

**TABLE 7:** Stress susceptibility and tolerance indices for photosynthetic rate and chlorophyll content.

**Genotypes**	**Photosynthetic rate**				**Chlorophyll content**			
	**SSI-T1**	**SSI-T2**	**STI-T1**	**STI-T2**	**SSI-T1**	**SSI-T2**	**STI-T1**	**STI-T2**
SITARA-16	4.149185	23.85456	0.558551	0.435918	−13.3327	1.624632	1.05528	0.896894
CIM-602	4.667792	5.125962	0.503374	0.878788	−16.928	−0.68509	1.070186	1.043478
CIM-779	−1.13976	−2.15814	1.121264	1.051033	12.20496	0.887972	0.949396	0.943646
6071/16	−0.21364	−4.15348	1.02273	1.098216	−12.01	0.150476	1.049795	0.99045
FH-326	2.091174	12.29666	0.777511	0.709224	23.31172	2.167315	0.903346	0.862454
FH-152	−0.65953	−7.14621	1.07017	1.168984	−0.78307	1.006127	1.003247	0.936147
IR-NIBGE-13	−0.18764	3.173445	1.019964	0.924958	−3.20611	1.856561	1.013293	0.882175
GH-HADI	−0.02664	−2.25964	1.002834	1.053433	−0.42166	1.175345	1.001748	0.925408
BS-2018	2.260667	−12.4802	0.759478	1.295117	5.977687	2.767657	0.975216	0.824353
NIAB-512	−0.77889	−7.86551	1.08287	1.185993	22.1498	−0.52255	0.908163	1.033163
NIAB-135	−0.51155	−5.5539	1.054426	1.131331	−9.08521	0.033597	1.037669	0.997868

*SSI-T1, stress susceptibility index under 15 dS/m salt stress; SSI-T2, stress susceptibility index under 20 dS/m salt stress; STI-T1, stress tolerance index under 15 dS/m salt stress; STI-T2, stress tolerance index under 20 dS/m salt stress.*

#### Transpiration Rate and Chlorophyll Content

Mostly in genotypes, salt stress decreased the transpiration rate. However, the least differences of salt stress for transpiration rate were observed in FH-326. Nevertheless, in 6071/16, transpiration rate was observed as increased under 15 dS/m salt stress. Thus, on the basis of SSI and STI, 6071/16 was found to be slightly tolerant under 15 dS/m but sensitive for severe 20 dS/m salt stress. Similarly, transpiration rate was significantly decreased in IR-NIBGE-13 under 20 dS/m salt stress. However, NIAB-512 and NIAB-135 maintained transpiration rate, which showed that they were least affected by salt stress (Table 6).

Chlorophyll content did not differ significantly in all the genotypes between salt-treated and non-treated plants before and after salt stress applied (chlorophyll content data before salt stress were not provided). This indicated that NaCl has no affect on the efficacy of light during photosynthesis.

#### Ionic Homeostasis (Na^+^/K^+^ Ratio)

NaCl treatment affected significantly ionic homeostasis in plant, i.e., Na^+^, K^+^, and Na^+^/K^+^ ratio. Na^+^ content was found to be increased in NaCl-treated plants especially under the increased salt concentration, i.e., 20 dS/m. Significant differences in Na^+^, K^+^, and Na^+^/K^+^ ratio was observed among the genotypes. In IR-NIBGE-13 and 6071/16, Na^+^ ion accumulated more in leaves as compared to K^+^ ion and increase in Na^+^/K^+^ ratio was observed. Na^+^ content was maintained in SITARA-16, GH-HADI, and FH-152.

K^+^ concentration significantly decreased in IR-NIBGE-13 and FH-152. The maximum value was recorded for K^+^ ion in CIM-779 under 20 dS/m salt stress condition. Moreover, K^+^ content was maintained in NIAB-135, NIAB-512, GH-HADI, and FH-326. Na^+^/K^+^ ratio significantly increased with the increase in salt concentration in IR-NIBGE-13, FH-152, FH-326, and 6071/16, whereas NIAB-512, NIAB-135, SITARA-16 CIM-602, and CIM-779 maintained the Na^+^/K^+^ ratio (Figure 6).

### Correlation Analysis Among Morphophysiological Traits

**FIGURE 7:**
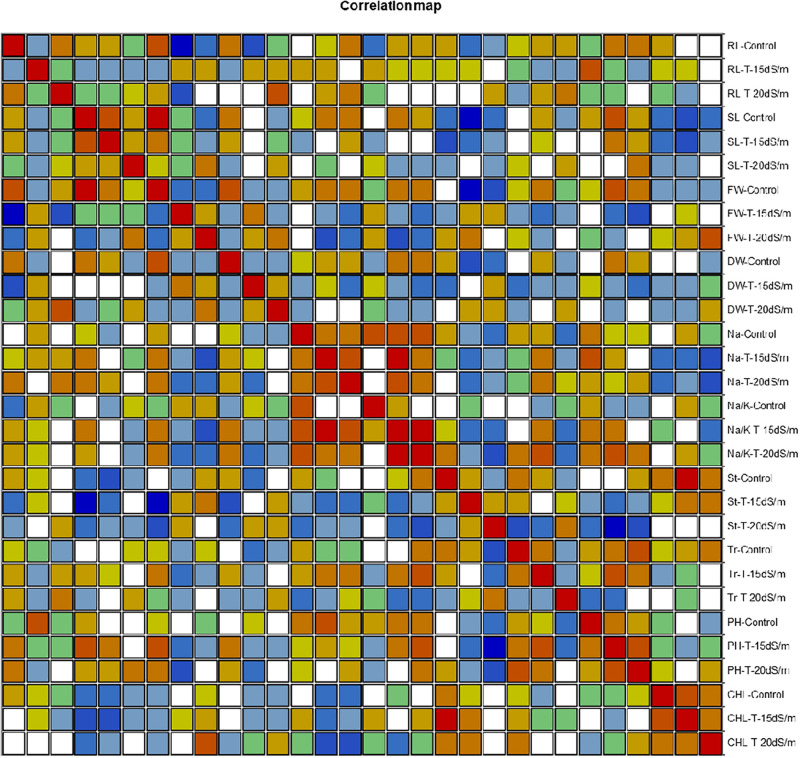
Correlation matrix showing Pearson’s correlation among morphophysiological traits in cotton genotypes under control and salt stress conditions. RL, root length; SL, shoot length; FW, fresh weight; DR, dry weight; Na, sodium content; Na/K, sodium to potassium ratio; St, stomatal conductance; Tr, transpiration rate; PH, photosynthetic rate; CHL, chlorophyll content.

Correlation (Pearson test) for morphophysiological traits under control and salt stress conditions was performed by using XLSTAT 2012.1.02 with 95% confidence interval (Figure 7 and Tables 8–10). Root length (RL) under control was positively correlated with fresh weight (FW) under control but negatively correlated with FW and dry weight (DW) under 15 dS/m salt stress, which showed that salt stress had significant effects on plant growth and biomass, whereas shoot length (SL) under control was positively correlated with SL (under 15 dS/m), FW (under control), and photosynthetic rate (PH-15 dS/m). SL under 15 dS/m was negatively correlated with stomatal conductance (St-control) and chlorophyll content (CHL under 15 dS/m). FW under control was positively correlated with DW (under control) but negatively correlated with St under salt stress. FW under 20 dS/m was negatively correlated with DW (under control), Na^+^ (under 15 dS/m), Na^+^/K^+^ ratio (under 15 dS/m), indicating that increase in Na^+^ and Na^+^/K^+^ ratio decreased the plant fresh weight under salt stress. Na^+^ ion concentration under 15 dS/m was positively correlated with Na^+^ ion under 20 dS/m, Na^+^/K^+^ ratio under 15 dS/m salt stress and PH under control condition. Na^+^ under 20 dS/m salt condition was positively correlated with Na^+^/K^+^ ratio under 15 dS/m salt stress condition. Similarly, Na^+^/K^+^ ratio under 15 dS/m was positively correlated with Na^+^/K^+^ ratio under 20 dS/m salt stress and also with Na^+^ treatments. Na^+^/K^+^ ratio under 20 dS/m was positively correlated with St-under 20 dS/m salt stress condition. Transpiration rate (Tr) under control and PH under 15 dS/m were positively correlated with PH under 20 dS/m, and CHL under control was positively correlated with CHL under 15 dS/m salt stress conditions.

**TABLE 8:** Correlation matrix for morphological traits under control and salt stress conditions.

**Variables**	**RL-Control**	**RL-15 dS/m**	**RL-20 dS/m**	**SL-Control**	**SL-15 dS/m**	**SL-20 dS/m**	**FW-Control**	**FW-15 dS/m**	**FW-20 dS/m**	**DW-Control**	**DW-15 dS/m**	**DW-20 dS/m**
RL-Control	**1**											
RL-15 dS/m	−0.382	**1**										
RL-20 dS/m	0.430	−0.148	**1**									
SL-Control	0.342	−0.273	−0.118	**1**								
SL-15 dS/m	0.297	−0.360	−0.137	**0.797**	**1**							
SL-20 dS/m	−0.103	−0.222	0.156	0.222	0.360	**1**						
FW-Control	**0.671**	−0.400	0.215	**0.851**	0.586	0.173	**1**					
FW-15 dS/m	**−0.850**	0.228	**−0.684**	−0.199	−0.119	−0.126	−0.569	**1**				
FW-20 dS/m^–1^	−0.471	0.211	−0.097	−0.470	−0.238	0.499	−0.432	0.337	**1**			
DW-Control	0.510	−0.256	−0.017	0.599	0.261	−0.219	**0.776**	−0.225	−0.302	**1**		
DW-15 dS/m	**−0.681**	0.279	−0.050	−0.033	−0.054	0.052	−0.330	0.511	0.201	−0.278	**1**	
DW-20 dS/m	−0.101	0.375	**0.621**	−0.367	−0.179	0.326	−0.241	−0.239	0.472	−0.328	0.312	**1**
Na-Control	−0.044	0.249	0.006	0.103	−0.276	−0.064	0.245	−0.096	−0.070	0.166	−0.253	−0.242
Na-15 dS/m	0.146	0.265	0.356	0.456	0.084	−0.161	0.452	−0.348	**−0.615**	0.228	0.111	0.086
Na-20 dS/m	0.451	−0.070	0.480	0.476	0.243	0.087	0.597	−0.526	−0.549	0.306	−0.408	0.004
Na/K-Control	−0.467	0.296	−0.137	−0.099	−0.322	0.148	−0.178	0.350	0.237	−0.223	0.134	−0.200
Na/K-15 dS/m	0.241	0.197	0.010	0.480	0.035	−0.389	0.529	−0.227	**−0.641**	0.478	−0.243	−0.371
Na/K-20 dS/m	0.399	0.116	−0.070	0.310	−0.052	−0.334	0.514	−0.415	−0.439	0.465	−0.512	−0.361
St-Control	0.216	0.124	−0.019	−0.444	**−0.611**	−0.239	−0.059	−0.231	0.249	0.243	−0.554	0.002
St-15 dS/m	−0.474	0.111	−0.060	**−0.851**	−0.527	−0.041	**−0.800**	0.306	0.523	**−0.626**	0.074	0.355
St-20 dS/m	−0.286	−0.062	0.256	−0.562	−0.323	−0.200	**−0.630**	0.343	0.078	−0.456	0.312	0.204
Tr-Control	0.103	−0.139	−0.346	0.048	0.099	0.200	0.148	−0.205	0.152	−0.053	−0.422	−0.257
Tr-15 dS/m	0.348	−0.346	0.253	0.293	0.117	−0.043	0.530	−0.512	−0.366	0.321	−0.288	−0.070
Tr-20 dS/m	0.284	−0.337	0.501	−0.252	0.062	0.383	−0.165	−0.296	0.083	−0.360	−0.243	0.315
PH-Control	−0.159	**0.673**	−0.184	0.275	−0.012	0.066	0.119	−0.095	−0.152	−0.089	0.144	0.060
PH-15 dS/m	0.486	−0.149	−0.185	**0.712**	0.507	−0.004	**0.751**	−0.434	−0.383	0.578	−0.211	−0.296
PH-20 dS/m	0.568	−0.238	0.022	0.360	0.334	0.475	0.567	**−0.635**	0.058	0.269	−0.597	−0.042
CHL-Control	0.264	0.154	−0.159	−0.470	−0.504	−0.351	−0.255	−0.100	0.168	0.024	−0.380	−0.203
CHL-15 dS/m	−0.095	0.184	−0.224	**−0.633**	**−0.776**	−0.272	−0.394	0.110	0.347	−0.066	−0.341	−0.203
CHL-20 dSm	0.027	−0.021	0.013	−0.600	−0.285	0.042	−0.365	−0.070	**0.640**	−0.267	−0.116	0.262

*Values in bold are different from 0 with a significance level alpha = 0.05. RL, root Length; SL, shoot length; FW, fresh weight; DW, dry weight; Na^+^/K^+^, sodium to potassium ration; St, stomatal conductance; Tr, transpiration rate; PH, photosynthetic rate; CHL, chlorophyll content.*

**TABLE 9:** Correlation matrix for physiological traits under control and salt stress conditions.

**Variables**	**Na-Control**	**Na-15 dS/m**	**Na-20 dS/m**	**Na/K-Control**	**Na/K-15 dS/m**	**Na/K-20 dS/m**	**St-Control**	**St-15 dS/m**	**St-20 dS/m**	**Tr-Control**	**Tr-15 dS/m**	**Tr-20 dS/m**	**PH-Control**	**PH-15 dS/m**	**PH-20 dS/m**	**CHL-Control**	**CHL-15 dS/m**	** CHL-20 dS/m**
Na-Control	**1**																	
Na-15 dS/m	0.469	**1**																
Na-20 dS/m	0.451	**0.755**	**1**															
Na/K-Control	**0.723**	0.098	0.084	**1**														
Na/K-15 dS/m	**0.731**	**0.812**	**0.699**	0.264	**1**													
Na/K-20 dS/m	**0.720**	0.541	0.479	0.089	**0.837**	**1**												
St-Control	0.346	−0.195	−0.087	−0.030	0.109	0.502	**1**											
St-15 dS/m	−0.220	−0.482	−0.508	−0.107	−0.548	−0.310	0.353	**1**										
St-20 dS/m	−0.441	−0.312	−0.241	0.048	−0.484	**−0.740**	−0.313	0.373	**1**									
Tr-Control	0.333	−0.152	−0.122	−0.012	0.085	0.539	0.435	0.211	**−0.710**	**1**								
Tr-15 dS/m	0.362	0.494	0.442	−0.270	0.500	**0.684**	0.273	−0.042	−0.570	0.540	**1**							
Tr-T-20 dS/m^–1^	−0.484	−0.327	0.132	−0.188	−0.547	−0.594	−0.204	0.135	0.575	−0.362	−0.360	**1**						
PH-Control	0.476	**0.614**	0.268	0.293	0.533	0.498	−0.005	−0.276	−0.591	0.261	0.145	−0.495	**1**					
PH-T-15 dS/m^–1^	0.138	0.347	0.198	−0.363	0.451	**0.646**	0.086	−0.529	**−0.871**	0.533	0.601	−0.520	0.410	**1**				
PH-T-20 dS/m	0.144	−0.028	0.217	−0.240	0.072	0.458	0.373	−0.219	**−0.782**	**0.709**	0.448	0.019	0.244	**0.670**	**1**			
CHL-Control	0.028	−0.509	−0.429	0.087	−0.187	0.093	0.595	0.117	0.074	0.121	−0.376	0.076	−0.137	−0.112	0.121	**1**		
CHL-15 dS/m	0.380	−0.420	−0.369	0.370	−0.053	0.244	**0.808**	0.404	−0.002	0.312	−0.132	−0.149	−0.055	−0.246	0.057	**0.798**	**1**	
CHL-20 dS/m	−0.158	**−0.696**	**−0.662**	−0.104	−0.596	−0.164	0.452	0.566	0.057	0.412	−0.094	0.081	−0.347	−0.131	0.200	0.566	0.521	**1**

*Values in bold are different from 0 with a significance level alpha = 0.05; Na+, Sodium concentration; Na + /K +, Sodium to Potassium ratio; St, Stomatal Conductance; Tr, Transpiration rate; PH, Photosynthetic rate; CHL, Chlorophyll content.*

**TABLE 10:** Stress susceptibility and tolerance Indices of Na^+^ and Na^+^/K^+^ ratio.

**Genotypes**	**Na^+^ content**				**Na + /K + ratio**			
	**SSI-T1**	**SSI-T2**	**STI-T1**	**STI-T2**	**SSI-T1**	**SSI-T2**	**STI-T1**	**STI-T2**
SITARA-16	0.341495	0.172787	1.139445	1.184181	0.147589	−0.1269	1.140432	0.845925
CIM-602	−0.02141	0.469071	0.991259	1.5	−0.66263	−0.2059	0.369501	0.75
CIM-779	8.847853	5.7499	4.612903	7.129032	4.489623	1.627089	5.271889	2.975596
6071/16	1.546711	1.516409	1.631579	2.616397	2.622059	2.172452	3.494896	3.637771
FH-326	7.524984	3.292024	4.072727	4.509091	4.299419	2.405675	5.090909	3.920949
FH-152	0.080237	−0.11057	1.032764	0.88214	2.204925	4.358455	3.097993	6.291996
IR-NIBGE-13	0.820384	0.967852	1.334993	2.031669	5.311725	5.100416	6.054122	7.192879
GH-HADI	−0.47396	−0.03025	0.806464	0.967757	0.079062	0.239124	1.075228	1.290343
BS-2018	−0.61577	1.383826	0.748558	2.475071	0.150801	0.327585	1.143488	1.397751
NIAB-512	1.253252	1.644566	1.511749	2.753004	0.808035	0.938074	1.768847	2.139
NIAB-135	3.738854	2.768775	2.526712	3.95134	2.073148	1.716422	2.972606	3.084063

SSI-T1, stress susceptibility index under 15 dS/m salt stress; SSI-T2, stress susceptibility index under 20 dS/m salt stress; STI-T1, stress tolerance index under 15 dS/m salt stress; STI-T2, stress tolerance index under 20 dS/m salt stress.

ANOVA for all morphological (Table 11) and physiological parameters explained in Table 12 indicated that shoot length, fresh and dry weights, and physiological parameters were highly significant except root length, which was non-significant under salt stress and non-stress conditions.

**TABLE 11:** ANOVA for morphological parameters.

	**Root length**	**Shoot length**	**Fresh weight**	**Dry weight**
R^2^	0.329	0.523	0.536	0.601
F	1.518	3.395	3.569	4.654
Pr > F	0.061	<0.0001	<0.0001	<0.0001

**TABLE 12:** ANOVA for physiological parameters.

	**Stomatal conduction (mmol m^–2^ s^–1^)**	**Transpiration rate (mmol m^–2^ s^–1^)**	**Photosynthetic rate (μmol m^–2^ s^–1^)**	**SPAD**	**Na^+^**	**K^+^**	**Na^+^/K^+^**
R^2^	0.646	0.576	0.743	0.583	0.961	0.974	0.932
F	5.658	4.208	8.954	4.323	75.241	117.646	42.148
Pr > F	<0.0001	<0.0001	<0.0001	<0.0001	<0.0001	<0.0001	<0.0001

### Cluster Analysis

**FIGURE 8:**
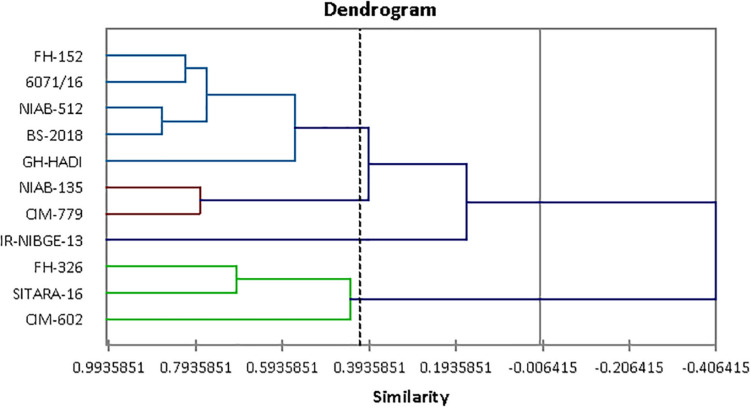
Tree diagram based on morphophysiological traits for different cotton genotypes under control and salt stress conditions.

Agglomerative hierarchical clustering of cotton genotypes was performed on the basis of morphophysiological traits under control and salt stress conditions (Figure 8). Cluster analysis grouped 11 genotypes into four clusters as shown in Table 13. Cluster I contained three genotypes followed by two, five, and one genotypes, respectively, in clusters II–IV.

**TABLE 13:** Distribution of cotton genotypes in different clusters on the basis of morphophysiological traits.

**Cluster**	**Genotypes**
I	SITARA-16, FH-326, CIM-602
II	CIM-779, NIAB-135
III	FH-152, NIAB-512, 6071/16, BS-2018, GH-HADI
IV	IR-NIBGE-13

### Principal Component Analysis

**FIGURE 9:**
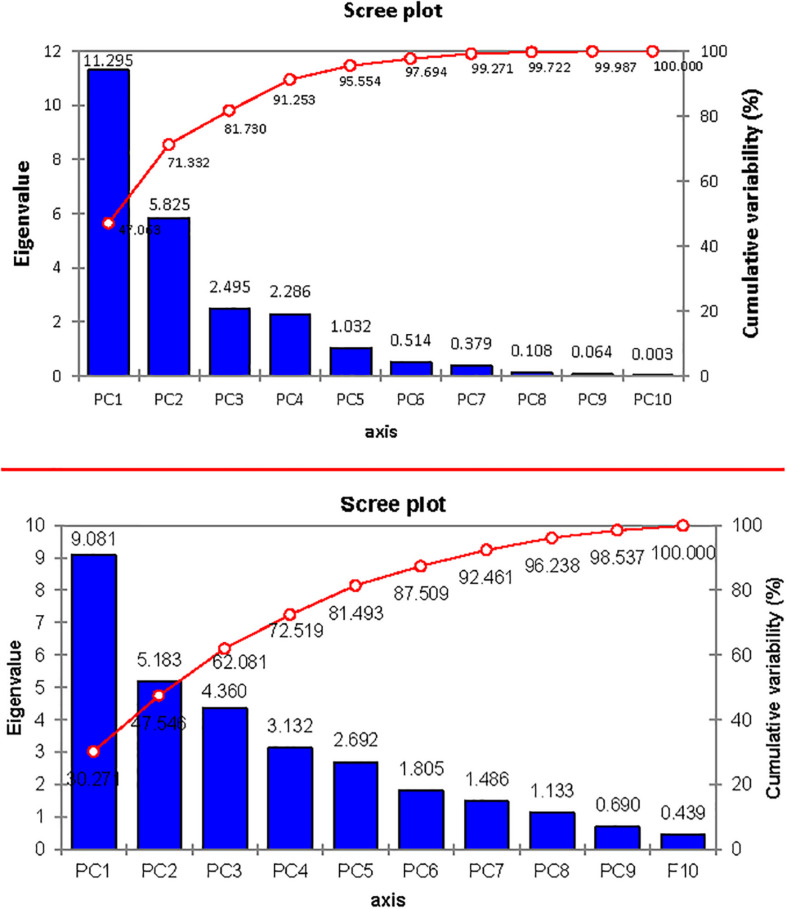
Scree plot between eigenvalues and principal components (PCs) under control and salt stress conditions.

The contribution of PC1 toward treatment was highest (47.06%) followed by PC2, PC3, PC4, and PC5, which contributed as 24.26, 10.39, 9.52, and 4.30%, respectively, as shown in Table 14. The first five PCs had eigenvalues > 1 and contributed 95.55% of the cumulative variability (Figure 9). On the basis of SSI and STI for morphophysiological traits, PC1 was mainly positively affected by SSI for FW, DW, Na^+^, Na^+^/K^+^ ratio, ST and STI for Na^+^/K^+^ ratio, Na + and PH, and vice versa. PC2 was positively affected by SSI and STI for Na^+^ and Na^+^/K^+^ ratio but negatively affected by FW-SSI (under 15 dS/m), FW-STI (under 20 dS/m), DW-STI (under 20 dS/m), PH-SSI and STI (under 15 and 20 dS/m), and ST-SSI under both stresses.

**TABLE 14:** Principal component analysis for morphophysiological traits under control and salt stress conditions in different cotton genotypes.

	**PC1**	**PC2**	**PC3**	**PC4**	**PC5**	**PC6**	**PC7**	**PC8**	**PC9**	**PC10**
Eigenvalue	11.295	5.825	2.495	2.286	1.032	0.514	0.379	0.108	0.064	0.003
Variability (%)	47.063	24.269	10.397	9.523	4.301	2.140	1.578	0.450	0.265	0.013
Cumulative%	47.063	71.332	81.730	91.253	95.554	97.694	99.271	99.722	99.987	100.000

**FIGURE 10:**
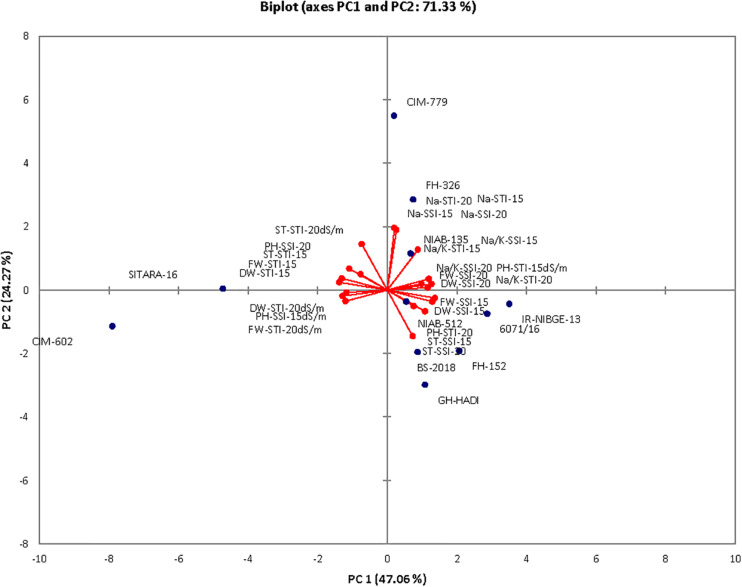
Biplot illustration of all the genotypes under control and salt stress conditions.

In biplot diagram, a vector was illustrated toward each genotypes and parameters from the origin to understand interrelationship between the genotypes along with traits (morphophysiological) and treatment’s SSI and STI (0, 15, 20 dS/m salt stress). Moreover PC1 and PC2 accounting for 47.06 and 24.26% were responsible for 71.33% variations among genotypes as shown in Figure 10. The biplot analysis indicated that CIM-779, CIM-602, and SITARA-16 were largely dispersed and away from the origin, which showed high variability for morphophysiological traits under salinity. The sensitive genotypes IR-NIBGE-13 and 6071/16 were very close to the traits FW-SSI and DW-SSI under 15 dS/m salt stress. The moderately tolerant genotypes NIAB-512, FH-152, BS-2018, and GH-HADI were close to the traits PH-STI (under 20 dS/m) and ST-SSI under both stress conditions. NIAB-135 was close to the traits Na^+^/K^+^ SSI and STI (under 15 dS/m). Genotypes positioned closer to the ideal genotypes like NIAB-135, FH-326, NIAB-512, GH-HADI, and FH-152 are preferable for biochemical selection along with poor performing genotypes, which are closed to each other but farthest away from tolerant genotypes, i.e., IR-NIBGE-13 and 6071/16 selected as sensitive genotypes for more biochemical analysis and confirmation.

### Biochemical Results

#### Lycopene

**FIGURE 11:**
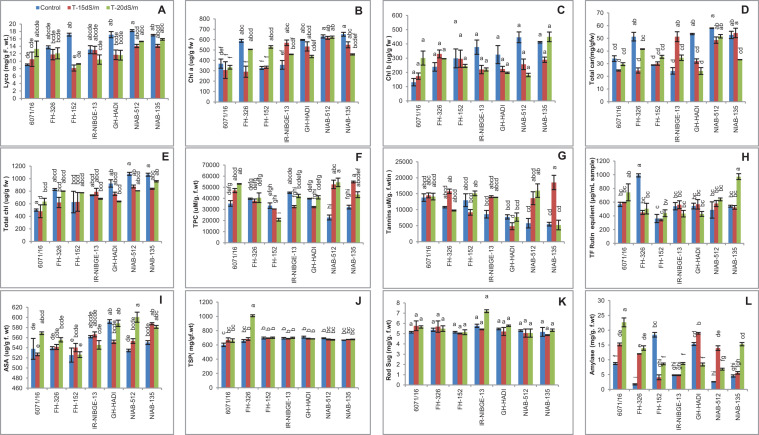
Comparison of panels **(A)** lycopene, **(B)** chlorophyll a, **(C)** chlorophyll b, **(D)** total carotenoids, **(E)** total chlorophyll, **(F)** total phenolic content, **(G)** tannins, **(H)** total flavonoids, **(I)** ascorbic acid, **(J)** total soluble proteins, **(K)** reducing sugars, and **(L)** amylase in cotton genotypes under stress and non-stress conditions.

Salt stress caused a marked decrease in lycopene content among the genotypes with the exception of 6071/16. In FH-152, lycopene content was significantly reduced under both stress conditions. However, lycopene content was maintained in the NIAB-135, NIAB-512, and FH-326 (Figure 11A).

#### Chlorophyll a

Chlorophyll a content was generally less affected by salt stress in most of the genotypes like NIAB-512, NIAB-132, and 6071/16. In IR-NIBGE-13, chlorophyll content increased under 15 dS/m but decreased with the increase of salt concentration. In contrast, chlorophyll content was significantly reduced in FH-236 under 15 dS/m but increased under 20 dS/m salt stress condition (Figure 11B).

#### Chlorophyll b

Chlorophyll b content was non-significant in all the genotypes. The decrease in chlorophyll b content was seen in GH-HADI, IR-NIBGE-13, and NIAB-512, but in 6071/16, chlorophyll content was increased under salt stress. In NIAB-135, chlorophyll b content was reduced under 15 dS/m but increased under 20 dS/m (Figure 11C).

#### Total Carotene

In general, total carotene content decreased in all the genotypes, except IR-NIBGE-13 in which the total carotene was more increased under 15 dS/m stress condition as compared to 20 dS/m. Carotene content was significantly decreased in FH-326 under 15 dS/m salt stress condition but maintained under 20 dS/m. However, in NIAB-512, FH-152, and 6017/16, carotene content showed least significant variations between stress and non-stress conditions. In NIAB-135, a significant decrease in carotene content under severe salt stress condition (20 dS/m) was observed. A significant decrease in carotene content was observed in GH-HADI under stress conditions (Figure 11D).

#### Total Chlorophyll

Salt stress had least significant difference in total chlorophyll content among the genotypes. Total chlorophyll content was non-significant in IR-NIBGE-13 and FH-152. However, a significant increase in NIAB-512 and 6017/16 under stress was observed. Salt-treated plants of GH-HADI had reduced chlorophyll content compared with that in control plants (Figure 11E).

#### Total Phenolic Contents

A significant increase in total phenolic content (TPC) in 6071/16, NIAB-512, and NIAB-135 was observed under salt stress conditions. However, the decrease in TPC content was observed in FH-152 under stress conditions. The salt-treated plants of FH-326 and GH-HADI were not significantly affected by the treatment and successfully maintained TPC. In IR-NIBGE-13, TPC content was significantly decreased under 15 dS/m, but with the increase in salt concentration, plants maintained TPC (Figure 11F).

#### Tannins

Generally, tannins were maintained in all the genotypes under control and stress conditions. Most genotypes showed non-significant increase for tannins except NIAB-512, which showed significant increase under salt stress conditions. In NIAB-135, tannins were significantly increased under 15 dS/m salt stress condition (Figure 11G).

#### Total Flavonoids

A general trend was seen in all the genotypes for total flavonoids. Mostly, the non-significant results were found under stress and non-stress conditions for total flavonoids. However, tolerant genotypes NIAB-135 showed significant increase in total flavonoids under 20 dS/m salt stress condition. The highest values recorded for total flavonoids were for NIAB-135 under stress and for FH-326 under control condition and lowest value for total flavonoids in FH-152 under control and 15 dS/m (Figure 11H).

#### Ascorbic Acid

For ascorbic acid (AsA) content, a significant increase in NIAB-512 and NIAB-135 under salt stress conditions was observed. In 6071/16, ascorbic acid was significantly increased under 20 dS/m salt stress condition. Ascorbic acid content was maintained in FH-326 and IR-NIBGE-13. Ascorbic acid content was significantly decreased in GH-HADI under 15 dS/m salt stress condition (Figure 11I).

### Total Soluble Protein

The genotypes did not show alleviatory effects of salt stress for total soluble protein. Mostly, the genotypes showed non-significant results for total soluble protein under stress and non-stress conditions. The maximum value was recorded for total soluble protein in FH-326 genotype under 20 dS/m, and minimum value was observed for 6071/16 under control condition (Figure 11J).

#### Reducing Sugar

Salt stress had no significant affect on reducing sugars in all the genotypes, as the general trend was observed among the genotypes. under stress and non-stress conditions (Figure 11K).

#### Amylase

The amylase content was significantly increased in 6071/16, FH-326, NIAB-512, and NIAB-135 under stress and non-stress conditions. In GH-HADI, a significant increase in amylase content under 15 dS/m was observed. However, amylase content was significantly reduced under 20 dS/m salt stress condition in GH-HADI. Amylase content was significantly decreased in FH-152, but in IR-NIBGE-13, a significant increase in amylase under 20 dS/m stress condition was observed (Figure 11L).

#### Catalase Activity

**FIGURE 12:**
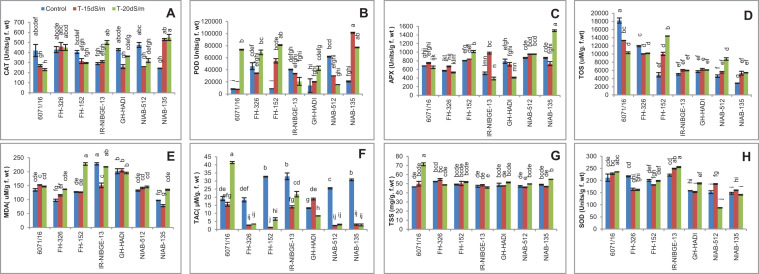
Comparison of panels **(A)** catalase, **(B)** peroxidase, **(C)** ascorbate peroxidase, **(D)** total oxidant status, **(E)** malondialdehyde, **(F)** total antioxidant capacity, **(G)** total soluble sugar, and **(H)** superoxide dismutase in cotton genotypes under stress and non-stress conditions.

For catalase activity in cotton leaves under salt stress, significant variations were found among the genotypes. The CAT activity was significantly increased in NIAB-135 under salt stress conditions. In contrast, CAT activity was reduced in 6071/16 and FH-152 under salt stress conditions. In NIAB-512 and GH-HADI, CAT activity was significantly increased under 20 dS/m compared with 15 dS/m salt stress condition in which it was significantly decreased. CAT activity was found to be increased under more salt concentrations of 20 dS/m among the genotypes in IR-NIBGE-13 and maintained in FH-326 under stress and non-stress conditions (Figure 12A).

#### Peroxidases Activity

For POD activity, a significant variation was found among cotton genotypes. In GH-HADI, FH-152, and NIAB-135, a significant increase in POD activity was observed under salt stress conditions. Similarly, a significant decrease in POD activity in some genotypes like NIAB-512 was observed. The highest value for POD activity was observed in NIAB-135 under 15 dS/m salt stress condition and lowest values in 6071/16 and FH-152 under control condition. In 6071/16, a significant increase in POD activity was observed under 20 dS/m salt condition. FH-326 exhibited significant increase under 20 dS/m and maintained under 15 dS/m stress condition. POD activity was maintained in IR-NIBGE-13 under stress and non-stress conditions (Figure 12B).

#### Ascorbate Peroxidase Activity

Ascorbate Peroxidase activity prevents the oxidative damage caused by H_2_O_2_ (Kohli et al., 2019). Plants under stress trigger the APX activity in the cell. Under the severe stress conditions, salt-treated plants of FH-152 and NIAB-135 had undergone oxidative stress; as a result, more activity of APX was seen under 20 dS/m. In contrast, APX activity was more increased in IR-NIBGE-13 under 15 dS/m compared to that in control and 20 dS/m. The genotypes 6071/16, NIAB-512, and FH-326 showed non-significant results for APX activity under salt stress conditions. GH-HADI showed significant decrease in APX activity under stress conditions (Figure 12C).

#### Total Oxidant Status

For TOS activity, a significant increase was observed in FH-152 and NIAB-512 under stress conditions except in 6071/16. The rest of the genotypes showed non-significant results for TOS under salt stress and non-stress conditions. In FH-152, TOS activity was significantly increased under stress (Figure 12D).

#### Malondialdehyde Content

The lipid peroxidation under salt stress was evaluated in terms of MDA content. In our results, salt stress caused low effect on lipid peroxidation, as most of the genotypes showed non-significant results. However, in FH-326, a significant increase in MDA content under stress conditions was observed. MDA content was significantly increased in NIAB-135 and IR-NIBGE-13 under 20 dS/m but significantly decreased under 15 dS/m salt stress condition. The maximum MDA content was recorded in FH-152 under 20 dS/m salt stress (Figure 12E).

#### Total Antioxidant Capacity

The tolerant genotypes, i.e.,FH-152, NIAB-512, and NIAB-135, had shown least activity of TAC under stress conditions as compared to control. However, in sensitive genotypes (i.e., 6071/16), TAC activity increased. The highest value for TAC was observed in 6071/16 under 20 dS/m and the lowest value in NIAB-135, NIAB-512, and FH-326 under stress conditions (Figure 12F).

#### Total Soluble Sugar

In general, a non-significant result for total soluble sugar was observed in all the genotypes except 6071/16, in which a significant increase in total soluble sugar was recorded under 20 dS/m salt stress condition (Figure 12G).

#### Superoxide Dismutase

The overproduction of reactive oxygen species (ROS) especially O_2_^–^ is mainly regulated by SOD (Kohli et al., 2019). More ROS generation leads to more activity of SOD enzyme. Similarly, in our results, more activity of SOD was seen under severe (20 dS/m) salt stress condition compared to control. However, the tolerant genotypes showed maintained activity of SOD under salt-treated and non-salt-treated plants. In contrast, SOD activity was found to be significantly increased in IR-NIBGE-13 under salt stress. In NIAB-512, SOD content was significantly increased under 15 dS/m salt stress, but under severe salt stress, SOD activity was reduced. In FH-326, SOD activity was significantly decreased under stress conditions. SOD content was significantly increased in GH-HADI under 20 dS/m salt stress but maintained under 15 dS/m salt stress condition. Similarly, it was maintained in FH-152 and NIAB-135 under salt stress and non-stress conditions (Figure 12H).

### Correlation Analysis Among Biochemical Traits

**FIGURE 13:**
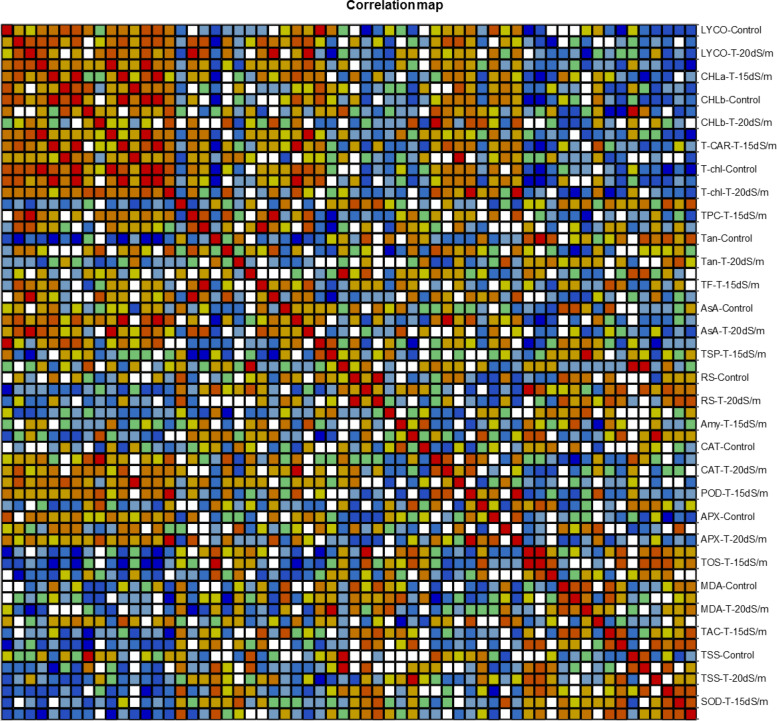
Correlation matrix showing Pearson’s correlation among biochemical traits in cotton genotypes under stress and non-stress conditions. LYCO, lycopene content; CHL-a, chlorophyll a; CHL-b, chlorophyll b; T-CAR, total carotenoids; T-chl, total chlorophyll; TPC, total phenolic content; Tan, tannins; TF, total flavonoids; AsA, ascorbic acid; TSP, total soluble protein; RS, reducing sugar; Amy, amylase; CAT, catalase; POD, peroxidase; APX, ascorbate peroxidase; TOS, total oxidant status; MDA, malondialdehyde; TAC, total antioxidant status; TSS, total soluble sugar; SOD, superoxide dismutase Cluster Analysis.

Correlation (Pearson test) for biochemical traits under control and salt stress conditions was performed by using XLSTAT 2012.1.02 with 95% confidence interval (Figure 13). Among all the genotypes under control and salt stress conditions, positive correlation with biochemical traits was expressed in bold form and negative correlation in bold form with negative sign as well. Thus, on this basis, significant correlation among genotypes under control, 15 dS/m, and 20 dS/m with biochemical traits related to stress tolerance was easily identified. In normal condition, LYCO was positively correlated with TSP and negatively correlated with TOS. Chlorophyll a (CHL-a) was positively correlated with total carotene (T-CAR) and negatively correlated with tannins (Tan). CHL-b was positively correlated with LYCO and total chlorophyll (T-CHL) and negatively with Tan and TOS. The negative correlation between TSP with TOS, amylase Amy with POD and Tan with SOD was observed under control conditions (Table 15).

**TABLE 15:** Correlation matrix for biochemical traits under normal conditions.

** Variables**	** LYCO-Control**	** CHLa-Control**	** CHLb-Control**	**T-CAR-Control**	** T-chl-Control**	** TPC-Control**	** Tan-Control**	** TF-Control**	** AsA-Control**	** TSP-Control**	** RS-Control**	**Amy-Control**	**CAT-Control**	**POD-Control**	**APX-Control**	**TOS-Control**	**MDA-Control**	**TAC-Control**	**TSS-Control**	**SOD-Control**
LYCO-Control	**1**																			
CHLa-Control	0.532	**1**																		
CHLb-Control	**0.789**	0.470	**1**																	
T-CAR-Control	0.514	**0.967**	0.347	**1**																
T-chl-Control	0.746	**0.898**	**0.810**	**0.815**	**1**															
TPC-Control	−0.472	−0.389	−0.350	−0.504	−0.432	**1**														
Tan-Control	−0.643	**−0.761**	**−0.879**	−0.633	**−0.943**	0.302	**1**													
TF-Control	−0.341	0.351	−0.349	0.321	0.059	0.355	0.084	**1**												
AsA-Control	0.123	0.274	0.183	0.190	0.274	0.515	−0.406	0.017	**1**											
TSP-Control	**0.819**	0.204	0.745	0.146	0.506	−0.007	−0.521	−0.337	0.389	**1**										
RS-Control	−0.069	−0.082	0.300	−0.223	0.095	0.650	−0.287	0.190	0.575	0.442	**1**									
Amy-Control	0.183	−0.447	−0.236	−0.345	−0.414	0.117	0.431	−0.572	0.178	0.275	−0.251	**1**								
CAT-Control	0.035	0.055	−0.319	0.287	−0.122	−0.343	0.311	0.129	−0.184	−0.011	−0.226	0.179	**1**							
POD-Control	0.231	0.425	0.512	0.383	0.537	−0.254	−0.509	0.366	−0.158	0.260	0.420	−**0.770**	0.186	**1**						
APX-Control	0.683	0.450	0.415	0.528	0.505	−**0.800**	−0.402	−0.555	−0.089	0.241	−0.650	0.294	0.120	−0.176	**1**					
TOS-Control	−**0.863**	−0.311	−**0.918**	−0.186	−0.664	0.189	0.713	0.448	−0.239	−**0.864**	−0.250	−0.095	0.386	−0.224	−0.432	**1**				
MDA-Control	−0.090	−0.346	0.159	−0.405	−0.151	0.556	−0.099	−0.299	0.684	0.433	**0.794**	0.247	−0.097	−0.025	−0.380	−0.194	**1**			
TAC-Control	0.213	−0.363	0.482	−0.491	−0.001	−0.127	−0.124	−0.488	−0.430	0.238	0.045	−0.053	−0.601	0.090	0.052	−0.536	−0.022	**1**		
TSS-Control	0.195	0.350	−0.127	0.321	0.169	0.244	0.073	0.710	−0.047	0.106	−0.041	−0.091	0.064	0.106	−0.183	−0.014	−0.451	−0.230	**1**	
SOD-Control	−0.737	−0.745	−0.634	−0.746	−**0.811**	0.629	**0.763**	0.352	−0.277	−0.382	0.290	−0.020	0.021	−0.066	−**0.870**	0.566	0.168	0.082	0.138	**1**

*Values in bold are different from 0 with a significance level alpha** = ** 0.05.*

Similarly, in 15 dS/m salt stress condition, LYCO was positively correlated with CHL-a, T-CAR, and T-Chl. However, CHL-a was negatively correlated with TOS and TSS. AsA was positively correlated with POD and negatively correlated with TOS, which showed that POD and AsA were involved in scavenging ROS. CAT was positively correlated with POD and negatively correlated with MDA, indicating that low activity of antioxidative enzymes like CAT and POD caused more oxidative damage in the form of lipid peroxidation. MDA (an indicator of oxidative damage) was positively correlated with TAC under 15 dS/m (Table 16). This correlation directly showed that with the increase in MDA, a relative amount of TAC also increased to cope with ROS under 15 dS/m salt stress.

**TABLE 16:** Correlation matrix for biochemical traits under 15 dS/m salt stress condition.

**Variables**	**LYCO-15 dS/m**	**CHLa-15 dS/m**	**CHLb-15 dS/m**	**T-CAR-15 dS/m**	**T-chl-15 dS/m**	**TPC-15 dS/m**	**Tan-15 dS/m**	**TF-15 dS/m**	**AsA-15 dS/m**	**TSP-15 dS/m**	**RS-15 dS/m**	**Amy-15 dS/m**	**CAT-15 dS/m**	**POD-15 dS/m**	**APX-15 dS/m**	**TOS-15 dS/m**	**MDA-15 dS/m**	**TAC-15 dS/m**	**TSS-15 dS/m**	**SOD-15 dS/m**
LYCO-15 dS/m	**1**																			
CHLa-15 dS/m	**0.760**	**1**																		
CHLb-15 dS/m	0.002	−0.176	**1**																	
T-CAR-15 dS/m	**0.756**	**0.866**	0.014	**1**																
T-chl-15 dS/m	**0.756**	**0.928**	0.203	**0.866**	**1**															
TPC-15 dS/m	0.618	0.282	−0.029	0.417	0.270	**1**														
Tan-15 dS/m	0.492	−0.028	0.231	0.375	0.059	0.672	**1**													
TF-15 dS/m	0.647	0.527	−0.722	0.324	0.251	0.452	0.129	**1**												
AsA-15 dS/m	0.706	0.725	0.236	**0.879**	**0.810**	0.319	0.359	0.162	**1**											
TSP-15 dS/m	−0.484	−0.038	0.338	−0.126	0.091	−**0.871**	−0.647	−0.645	−0.028	**1**										
RS-15 dS/m	−0.231	−0.595	−0.335	−0.629	−0.719	−0.210	0.113	0.228	−0.650	−0.258	**1**									
Amy-15 dS/m	0.083	−0.011	−0.399	−0.442	−0.162	0.103	−0.405	0.546	−0.451	−0.317	0.374	**1**								
CAT-15 dS/m	0.297	−0.121	0.677	0.219	0.136	0.318	0.666	−0.304	0.548	−0.166	−0.176	−0.451	**1**							
POD-15 dS/m	0.262	0.227	0.582	0.554	0.446	0.330	0.443	−0.374	**0.766**	0.033	−0.710	−0.674	**0.782**	**1**						
APX-15 dS/m	0.221	0.559	−0.250	0.590	0.462	−0.029	−0.002	0.163	0.184	0.167	−0.265	−0.364	−0.474	−0.097	**1**					
TOS-15 dS/m	−0.728	−**0.922**	−0.175	−**0.837**	−**0.984**	−0.157	0.018	−0.254	−**0.838**	−0.169	0.691	0.148	−0.149	−0.439	−0.392	**1**				
MDA-15 dS/m	−0.200	0.142	−0.631	−0.315	−0.098	−0.502	−**0.812**	0.395	−0.419	0.270	0.277	0.662	−**0.829**	−**0.788**	0.112	0.023	**1**			
TAC-15 dS/m	−0.041	0.114	−**0.834**	−0.173	−0.203	−0.315	−0.445	0.620	−0.176	−0.047	0.426	0.504	−0.526	−0.593	−0.055	0.088	**0.790**	**1**		
TSS-15 dS/m	−0.500	−**0.860**	0.408	−0.737	−0.701	−0.398	0.119	−0.511	−0.550	0.170	0.670	−0.022	0.285	−0.221	−0.509	0.675	−0.167	−0.178	**1**	
SOD-15 dS/m	−0.056	−0.004	−0.626	0.133	−0.241	−0.102	0.213	0.320	−0.183	−0.156	0.445	−0.257	−0.412	−0.392	0.609	0.259	0.151	0.369	−0.050	**1**

*Values in bold are different from 0 with a significance level alpha = 0.05.*

LYCO under 20 dS/m was positively correlated with TF and AsA and negatively correlated with MDA. CHL-a and CHL-b were positively correlated with T-CAR and TF under severe salt stress (20 dS/m). Amy was positively correlated with TSS, hence showed that sugar has some protective role under salt stress. SOD was negatively correlated with T-Chl content, explaining more SOD activity due to more ROS production in cell, which had effect on other parameters in the cell like T-Chl content (Table 17).

**TABLE 17:** Correlation matrix for biochemical traits under 20 dS/m salt stress condition.

**VariablesLYCO-20 dS/m**	**CHLa-20 dS/m**	**CHLb-20 dS/m**	**T-CAR-20 dS/m**	**T-chl-20 dS/m**	**TPC-20 dS/m**	**Tan-20 dS/m**	**TF-20 dS/m**	**AsA-20 dS/m**	**TSP-20 dS/m**	**RS-20 dS/m**	**Amy-20 dS/m**	**CAT-20 dS/m**	**POD-20 dS/m**	**APX-20 dS/m**	**TOS-20 dS/m**	**MDA-20 dS/m**	**TAC-20 dS/m**	**TSS-20 dS/m**	**SOD-20 dS/m**
LYCO-20 dS/m**1**																			
CHLa-20 dS/m0.093	**1**																		
CHLb-20 dS/m0.461	−0.360	**1**																	
T-CAR-20 dS/m0.329	**0.826**	−0.209	**1**																
T-chl-20 dS/m0.532	0.471	0.650	0.451	**1**															
TPC-20 dS/m0.735	−0.149	0.003	0.235	−0.106	**1**														
Tan-20 dS/m-0.315	0.294	−0.629	0.465	−0.381	0.025	**1**													
TF-20 dS/m**0.848**	−0.209	**0.799**	0.044	0.600	0.462	−0.382	**1**												
AsA-20 dS/m**0.808**	0.109	−0.009	0.154	0.118	**0.763**	−0.301	0.454	**1**											
TSP-20 dS/m-0.171	0.167	0.091	0.270	0.170	−0.159	−0.197	−0.269	−0.263	**1**										
RS-20 dS/m-0.405	−0.378	−0.203	−0.326	−0.484	0.072	0.052	−0.362	−0.287	−0.056	**1**									
Amy-20 dS/m0.311	−0.750	0.625	−0.333	−0.046	0.349	−0.186	0.586	0.004	0.071	−0.083	**1**								
CAT-20 dS/m0.173	0.084	0.478	0.006	0.541	−0.058	−0.630	0.206	−0.006	0.277	0.390	−0.162	**1**							
POD-20 dS/m-0.074	−0.403	0.683	−0.375	0.286	−0.450	−0.338	0.340	−0.402	0.223	−0.444	0.610	−0.089	**1**						
APX-20 dS/m0.555	0.265	0.652	0.233	**0.847**	−0.098	−0.232	0.749	0.154	−0.292	−0.606	0.103	0.191	0.407	**1**					
TOS-20 dS/m-0.437	0.198	−0.183	0.232	−0.065	−0.498	0.584	−0.285	−0.572	0.207	−0.481	0.072	−0.652	0.467	0.060	**1**				
MDA-20 dS/m-**0.856**	0.023	−0.511	−0.336	−0.450	−0.679	0.330	−0.722	−0.612	−0.288	0.445	−0.549	−0.109	−0.167	−0.336	0.204	**1**			
TAC-20 dS/m-0.133	−**0.768**	−0.036	−0.418	−0.669	0.352	0.353	0.062	−0.154	−0.318	0.435	0.641	−0.433	0.058	−0.393	0.053	0.058	**1**		
TSS-20 dS/m0.272	−0.696	0.339	−0.378	−0.259	0.352	0.068	0.507	0.126	−0.327	−0.233	**0.857**	−0.560	0.489	0.123	0.209	−0.355	0.733	**1**	
SOD-20 dS/m-0.670	−0.723	−0.074	−0.664	−0.665	−0.250	0.153	−0.361	−0.624	−0.110	0.719	0.294	−0.083	0.145	−0.542	0.051	0.567	0.727	0.264	**1**

*Values in bold are different from 0 with a significance level alpha = 0.05.*

**FIGURE 14:**
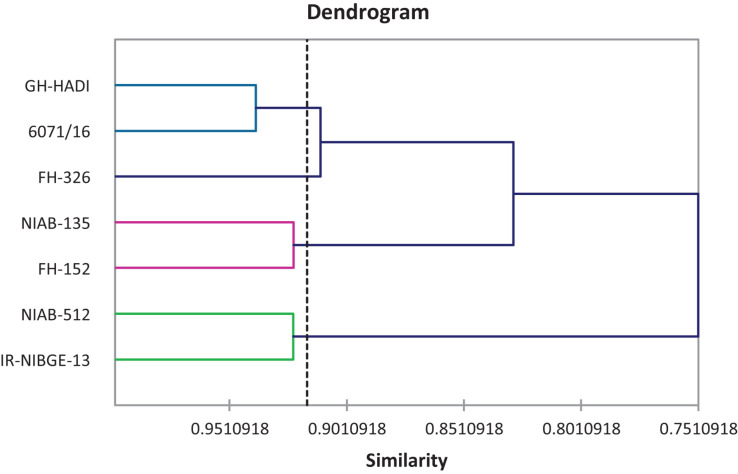
Tree diagram based on biochemical traits for different cotton genotypes under control and salt stress conditions.

Agglomerative hierarchical clustering of cotton genotypes was performed on the basis of biochemical traits under control and salt stress conditions (Figure 14). Cluster analysis grouped seven genotypes into four clusters as shown in Table 18. Cluster I contained one genotype followed by two, two, and two genotypes, respectively, in clusters II, III, and IV.

**TABLE 18:** Distribution of cotton genotypes in different clusters.

**Cluster**	**Genotypes**
I	FH-326
II	GH-HADI, 6071/16
III	FH-152, NIAB-135
IV	IR-NIBGE-13, NIAB-512

### Principal Component Analysis

**FIGURE 15:**
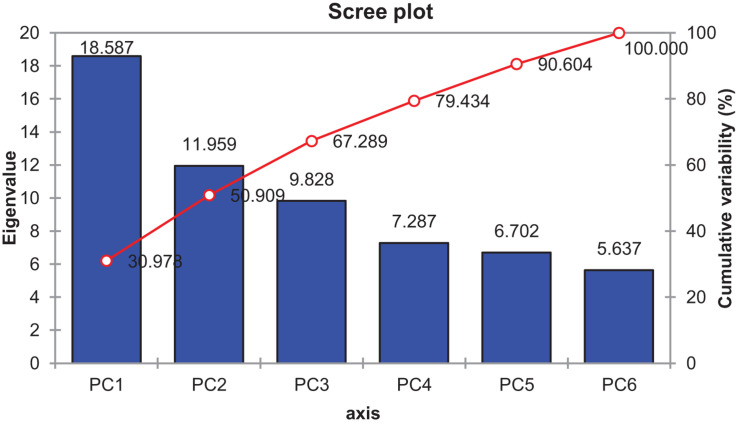
Scree plot between eigenvalues and principal components (PCs) under control and salt stress conditions.

**FIGURE 16:**
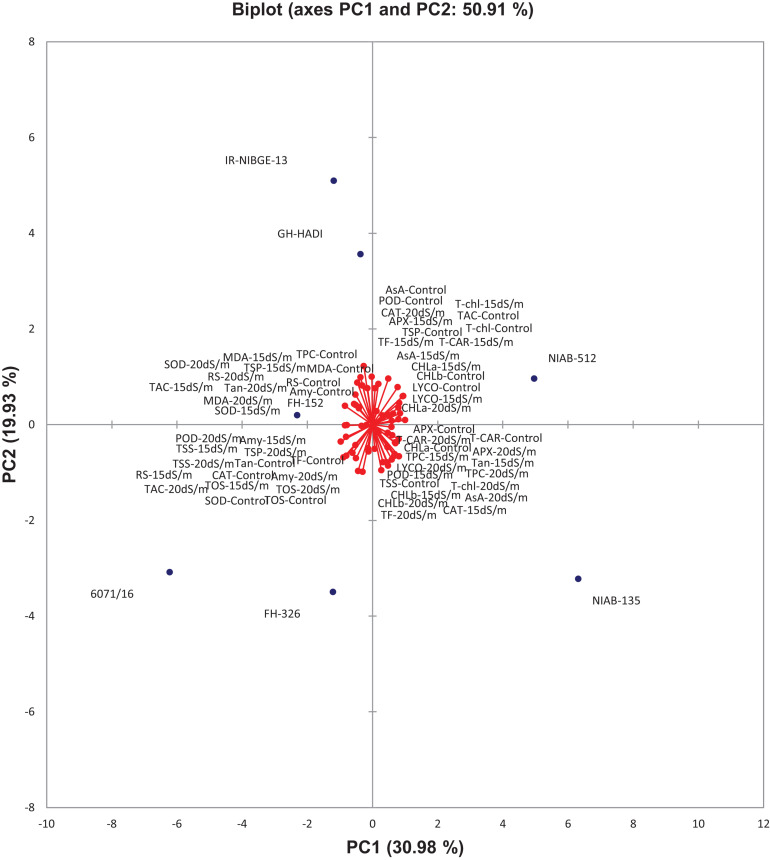
Biplot illustration of all the genotypes under control and salt stress conditions.

The contributed cumulative variability of PC1 toward treatment was highest (30.978%) followed by PC2, PC3, PC4, PC5, and PC6, which contributed as 50.909, 67.289, 79.434, 90.604, and 100.000%, respectively (Figure 15). Thus, the highly contributed PCs are shown in Table 19. The PC1 had mostly positive loading for biochemical traits except for TPC, Tan, TF, RS, Amy, CAT, TOS, MDA, and SOD (under control); TSP, RS, Amy, TOS, MDA, TAC, TSS, and SOD (under 15 dS/m); and Tan, TSP, RS, Amy, TOS, MDA, TAC, TSS, and SOD (under 20 dS/m). The PC2 was mostly negative affected except for CHL-a, T-CAR, Tan, TF, CAT, APX, TOS, TSS, and SOD (under control); CHL-b, TPC, Tan, RS, Amy, CAT, POD, APX, TOS, and TSS (under 15 dS/m); and LYCO, CHL-b, T-CAR, T-chl, TPC, TF, AsA, TSP, Amy, POD, APX, TOS, TAC, and TSS (under 20 dS/m). In biplot diagram, a vector was illustrated toward each genotypes and parameters from the origin to understand interrelationship between the genotypes along with traits (biochemical) and treatments (0, 15, and 20 dS/m salt stress conditions). Moreover, PC1 and PC2 were responsible for 50.91% variations among genotypes as shown in Figure 16.

**TABLE 19:** Principal component analysis for biochemical traits under control and salt stress conditions in different cotton genotypes.

	**PC1**	**PC2**	**PC3**	**PC4**	**PC5**	**PC6**
Eigenvalue	18.587	11.959	9.828	7.287	6.702	5.637
Variability (%)	30.978	19.931	16.380	12.145	11.171	9.396
Cumulative%	30.978	50.909	67.289	79.434	90.604	100.000

## Discussion

Seed germination, seedling emergence, and plant growth are affected by salinity stress in cotton (Zhong and Läuchli, 1993), although cotton is considered as the second most salt-tolerant crop, barely with salinity threshold at 7.7 dS/m, but increase in salt concentration adversely affects cotton yield (Higbie et al., 2010). Salt stress causes water deficiency in roots and imposes drought to the plant. Many physiological (stomatal conductance and photosynthetic activity) and metabolic changes occur under salt stress. Initially, it causes osmotic stress, which interrupts the physiological functioning followed by ion toxicity and oxidative stress (Higbie et al., 2010). However, plants developed different physiological and biochemical defense mechanisms in order to maintain their cellular and metabolic activities, in which most important mechanism is Na^+^ compartmentalization (Peng et al., 2016). The maintenance of ion homeostasis especially Na^+^ sequestration is key mechanism to survive under salinity along with activation of antioxidant enzymes (Gupta and Huang, 2014).

In our experiment, initial screening for salt tolerance under 0, 7.5, and 15 dS/m was done by seed germination and radical elongation (growth chamber) test, and then, selected sensitive and tolerant genotypes were transferred into sand pots (glass house) for further screening at seedling stage under 15 and 20 dS/m salt stress conditions. With the help of these two screening procedures, it showed that genotypes possessed different genetic diversity for salt tolerance. Thus, the evidence of these differences was based on plant fresh and dry weights, photosynthetic rate, Na^+^/K^+^ ratio, and biochemical and stress markers (SOD, POD, APX, CAT, MDA, TOS, amylase, and AsA).

Results of the current study showed that in the first experiment, salt stress negatively affected seed germination parameters, i.e., growth index and growth energy, which is highly retarded by salt. As the cotton germination is very a critical stage, some genotypes performed well even at this stage of severe salt stress, i.e., NIAB-512, NIAB-135, and FH-152. Thus, the salt tolerance ability of plants is genotypic dependent. Plants that showed tolerance under germination stage performed relatively better under increased salt concentrations at their early growth stages.

Salt stress reduces the dry weight and biomass accumulation (Higbie et al., 2010). A significant decrease in fresh and dry weights was also observed in our experiment. NaCl treatment significantly reduced plant biomass in two genotypes IR-NIBGE-13 and BS-2018. A similar trend was also noted in other genotypes. However, NIAB-135, NIAB-512, and GH-HADI had the least difference in fresh weight between control and NaCl-treated plants. Plant fresh weight was significantly reduced in 6071/16. Thus, a reduction in plant biomass was found to be reliable method for the determination of salt stress tolerance in cotton, as the reduction in sensitive genotypes was more than tolerant genotypes. The root and shoot lengths showed non-significant change. On the basis of STI, CIM-602, GH-HADI, and FH-326 found to be tolerant genotypes for root length under stress conditions. Similarly, CIM-602, NIAB-512, NIAB-135, CIM-779, and FH-326 showed more STI values for shoot length. However, the comparison between the tolerant and susceptible genotype was done with the help of plant fresh and dry weights.

Chlorophyll fluorescence does not change under NaCl condition (Higbie et al., 2010). Similar results were also found in our experiment. Chlorophyll content was observed to be non-significant in all the genotypes between salt-stress-treated and non-treated plants. Under salt stress, closure of stomata and reduction in photosynthetic rate was reported (Brugnoli and Lauteri, 1991). Stomatal closure reduced the CO_2_ uptake and limited the activity of Rubisco, which ultimately reduced the photosynthesis. In this way, salinity reduced the plant growth and cotton yield (Ahmad and Prasad, 2011). In our experiment, stomatal conductance was significantly decreased in IR-NIBGE-16, 6071/16, GH-HADI, and FH-152 but maintained in NIAB-135, NIAB-512, SITARA-16, and FH-326. Photosynthetic rate was maintained in all the genotypes with the exception of SITARA-16. According to SSI, IR-NIBGE-13 was found to be more sensitive under 20 dS/m salt stress condition. Transpiration rate decreased under stress conditions. However, the tolerant cotton genotypes have more transpiration rate under salinity than sensitive genotypes (Dong et al., 2020). In our study, most genotypes showed decrease in transpiration rate under stress conditions. Transpiration rate remained the same in FH-326 under stress and non-stress conditions. Thus, on the basis of SSI and STI, 6071/16 found to be tolerant under 15 dS/m but sensitive under 20 dS/m salt stress. Similarly, transpiration rate was significantly decreased in IR-NIBGE-13 under 20 dS/m salt stress. In NIAB-512 and NIAB-135, maintained transpiration rate under stress conditions was observed.

Salinity stress significantly increased Na^+^/K^+^ ratio in cotton (Cramer et al., 1987; Ibrahim et al., 2019). NaCl treatment affected significantly ionic homeostasis in plant, i.e., Na^+^, K^+^, Cl, Zn, Mn, N, and Na^+^/K^+^ ratio (Cramer et al., 1987; Ashraf and Ahmad, 2000; Munns and Tester, 2008; Higbie et al., 2010). Na^+^ content found to be increased in NaCl-treated plants compared to non-NaCl-treated plants. Moreover, significant differences in Na^+^, K^+^, and Na^+^/K^+^ ratio was also observed among the genotypes. In IR-NIBGE-13 and 6071/16, Na^+^ ion accumulated more in leaves as compared to K^+^ ion under stress conditions, and increased in Na^+^/K^+^ ratio was also observed. Na^+^ content remained the same in SITARA-16, GH-HADI, and FH-152 under stress and non-stress conditions. K^+^ concentration significantly decreased in IR-NIBGE-16 and FH-152. Na^+^ accumulation in leaves observed a linear increase up to 20 dS/m. Contrary, K^+^ concentrations remained stable in all the genotypes except FH-152 and IR-NIBGE-13. Tolerant genotypes had low Na^+^/K^+^ ratio as compared to susceptible genotypes. The maximum value recorded for K^+^ ion in CIM-779 at 20 dS/m salt stress condition. Na^+^/K^+^ ratio was significantly increased in IR-NIBGE-16, FH-152, FH-326, and 6071/16. Na^+^/K^+^ ratio was maintained in NIAB-512, NIAB-135, SITARA-16 CIM-602, and CIM-779 under stress conditions.

Salt stress is known to cause the oxidative stress in plants, which results in oxidation of many cellular compounds by increased production of ROS and lipid peroxidation of plasma membrane (Ibrahim et al., 2019). Natural enzymatic antioxidants act as scavengers against these reactive oxygen species like SOD (Gupta and Huang, 2014). SOD converts the O^2–^ into H_2_O_2_ and O_2_. On the other hand, in peroxisomes, H_2_O_2_ is converted into H_2_O and O_2_ by the action of CAT. H_2_O_2_ can also be reduced by the action of peroxidase, as it provides various electron donors for the reduction in ROS (Alscher et al., 2002). Under normal conditions, in photosynthesis, the exited electrons move in electron transport chain (ETC) in thylakoid membrane, converting the oxidize form of NADP + into its reduced NADPH form at photosystem I (PSI). However, under stress, ETC overloaded with the electrons from the photolysis caused the overproduction of ROS, i.e., O^2–^. This reactive oxygen species converted into H_2_O_2_ by the action of SOD and rapidly detoxify by APX and CAT reaction into stable molecules, i.e., H_2_O and O_2_ (Ahammed et al., 2018; Kohli et al., 2019). Thus, the decrease in CO_2_ fixation leads to ROS production and how physiological imbalance leads to oxidative stress. Salt-treated plants of IR-NIBGE-13 and 6071/16 limit the stomatal conductance and photosynthetic activity encountered the more oxidative stress.

In plant, SOD and POD content is positively corelated with salt stress tolerance (Gupta and Huang, 2014). Proteomics studies revealed that stressful environment can affect the activity of antioxidants (Zhao et al., 2013). More SOD activity under salinity stress showed that plant is adapted to adverse environmental conditions. Salinity stress caused the reduction in photosynthetic activity, which increased the reactive oxygen species production. Thus, the SOD and POD enzymes are found to be increased in order to detoxify ROS and overcome its generation in plants (Ozgur et al., 2013). In our study, it was found that SOD content significantly increased in IR-NIBGE-13 under salt stress. In NIAB-512, SOD content was significantly increased under 15 dS/m salt stress and decreased in FH-326, under stress conditions. SOD content was significantly increased in GH-HADI under 20 dS/m salt stress. Similarly, it was maintained in FH-152 under salt stress and non-stress conditions. For POD activity, a significant increase was observed under salt stress conditions in GH-HADI, NIAB-135, and FH-152. Similarly, a significant decrease in POD activity in some genotypes like NIAB-512 was observed. In 6071/16 genotype, a significant increase in POD activity was observed under 20 dS/m salt condition. POD activity was maintained in IR-NIBGE-13 under stress and non-stress conditions.

Ascorbate peroxidase and CAT activity is also reported to be increased in cotton plants when exposed to salinity in order to cope with ROS production and protect plant from oxidative damage (Sekmen et al., 2014; Abdelraheem et al., 2019). In our experiment, increased level of APX was observed in tolerant genotypes as FH-152 and NIAB-135 under 20 dS/m salt stress. GH-HADI showed significant decrease in APX activity under stress conditions. The catalase activity was significantly increased in NIAB-135 and reduced in 6071/16 and FH-152 under salt stress conditions. In NIAB-512 and GH-HADI, CAT activity was significantly increased under 20 dS/m compared with that under 15 dS/m salt stress condition, in which it was significantly decreased. CAT activity increased significantly under 20 dS/m in IR-NIBGE-13. APX and CAT, together with SOD, performed well in scavenging process of oxidants (Abdelraheem et al., 2019).

According to proteomic studies, CAT activity was decreased in prolonged period of salt stress due to decrease in CAT levels (Zhao et al., 2013). Similar behaviors were also reported for MDA content, as with increase in salt concentration and stress duration, MDA and CAT activity reduced. However, in our experiment, MDA content was increased under severe salt stress condition, as MDA is a product of lipid peroxidation of cellular membrane under stress condition (Moussouraki et al., 2019). In NIAB-135 and IR-NIBGE-13, MDA content was significantly increased under 20 dS/m but significantly decreased under 15 dS/m salt stress conditions. In FH-326, a significant increase in MDA content under stress conditions was observed. The more MDA accumulation indicated higher lipid peroxidation due to salinity stress (Meloni et al., 2003). Antioxidant activity varies from plant to plant. Scientists suggested that there can be many factors for this difference in antioxidant activities in genotypes, which includes difference in stomata closure degree or other responses that change the degree of CO_2_ fixation or may be the difference that cause the photo-inhibition (Munns and Tester, 2008).

Accumulation of carbohydrates (like sugars) under stress conditions is the one of most important plant response mechanisms in order to attain stress tolerance by osmo-protection, carbon storage, and working against ROS (Kerepesi and Galiba, 2000; Parida et al., 2004). In our experiment, a significant increase was observed in total soluble sugar and amylase in NIAB-135 and 6071/16. However, non-significant result was found for reducing sugars.

The activity of other non-enzymatic antioxidants such as lycopene, AsA, tannins, TOS, and total phenolic content was significantly increased under salt stress conditions in tolerant genotypes, i.e., NIAB-135, NIAB-512, and FH-152. The lesser accumulation of MDA content and higher activity of enzymatic antioxidants such as SOD, POD, and APX under stress-treated plants of NIAB-135, NIAB-512, and FH-152 indicated that these genotypes had adaption capacity for salinity stress in comparison with sensitive genotypes, i.e., IR-NIBGE-13 and 6071/16. The bioplot analysis revealed that NIAB-135, 6071/16, FH-326, IR-NIBGE-13, and GH-HADI were highly dispersed from the origin point and had high genetic variability, whereas FH-152 was very close to the traits, i.e., Amy, RS, TPC, and MDA (under control); SOD, TAC, MDA, and TSP (under 15 dS/m); and MDA, Tan, SOD, and RS (under 20 dS/m). Similarly, NIAB-512 was closed to the LYCO, TAC, TSP, AsA, and CHL-b (under control); LYCO, CHL-a, TF, APX, T-chl, and AsA (under 15 dS/m); and CAT, TSP, and CHL-a (under 20 dS/m).

## Conclusion

Salinity tolerance mechanism involves many complex responses at cellular, metabolical, physiological, biochemical, and molecular levels. As an overall conclusion, the present study revealed that cotton genotypes had significant variations for morphophysiological and biochemical traits under salinity stress. The observed salt tolerance was corelated with plant biomass maintenance (morphological), photosynthetic rate, and ionic homeostasis (K^+^/Na^+^ ratio, physiological) and biochemical stress marker regulations. From the data presented, after a series of experiments, we found that out of the tested genotypes, NIAB-135, NIAB-512, and FH-152 could be used to develop breeding strategies for improvement of salinity tolerance in cotton.

## Data Availability Statement

The raw data supporting the conclusions of this article will be made available by the authors, without undue reservation.

## Author Contributions

WM did the overall execution of the experiment, analytical work, collection of data after morpho-physiological and biochemical analysis of leaves, organization of resulting data, and writing up and revision of manuscript. AH contributed to the planning, designing and finalization of basic idea of experiment and overall supervision during analytical work, carried out the statistical analysis of data using XL-STAT software, did the presentation of resulting data in the form of graphs, and revised and finalized the manuscript. MK did the arrangement and provision of cotton seeds, contributed in study basic idea and planning of glass house experiment, and revised and finalized the manuscript. All authors contributed to the article and approved the submitted version.

## Conflict of Interest

The authors declare that the research was conducted in the absence of any commercial or financial relationships that could be construed as a potential conflict of interest.
